# Untargeted Metabolomics Reveals Seasonal and Tissue‐Specific Metabolic Shifts in *Holothuria cinerascens*

**DOI:** 10.1002/cbdv.202501499

**Published:** 2025-12-05

**Authors:** Cassandra Upton, Gerhard Prinsloo, Paul Steenkamp, Moses Okpeku

**Affiliations:** ^1^ Discipline of Genetics, School of Life Sciences University of KwaZulu‐Natal Durban South Africa; ^2^ Department of Agriculture and Animal Health University of South Africa (UNISA), Science Campus Johannesburg South Africa; ^3^ Research Centre for Plant Metabolomics, Department of Biochemistry University of Johannesburg Auckland Park South Africa

**Keywords:** *Holothuria cinerascens*, holothurian, metabolomics, natural products, nuclear magnetic resonance (NMR) spectroscopy, ultra performance liquid chromatography quadruple time‐of‐flight mass spectrometry (UPLC–QTOF–MS)

## Abstract

Sea cucumbers are valuable marine invertebrates known for their biologically active compounds with health‐promoting properties. However, research has largely focused on select high‐value species, overlooking others with promising bioactive potential. This study presents the first untargeted metabolomic analysis of *Holothuria cinerascens* from KwaZulu‐Natal, South Africa, using ^1^H‐nuclear magnetic resonance (NMR) and ultra performance liquid chromatography quadruple time‐of‐flight mass spectrometry (UPLC–QTOF–MS) to assess metabolic and seasonal variability across three tissues: body wall, gonad and gut/mesentery. The body wall exhibited the highest metabolite diversity, with elevated levels of amino acids and potential triterpene glycosides, likely linked to stress or defence responses, whereas the gut/mesentery showed higher levels of sugars (galactose, xylose) and glycerol, possibly reflecting energy metabolism, diet or microbial activity. The gonad showed the lowest overall metabolite abundance but the highest levels of betaine and pyruvate. Seasonal differences were most pronounced in the gonad and gut/mesentery, likely related to reproductive activity and increased food availability. Compound identification was limited by structural isomerism and gaps in holothurian literature and databases, leaving several metabolites unidentified. These findings highlight the untapped potential of *H. cinerascens* and underscore the need to expand holothurian research. Future studies should prioritise compound characterisation and examine environmental influences on metabolite profiles to inform sustainable cultivation practices and natural product discovery.

## Graphical abstract

Untargeted metabolic profiling of three body tissues from *Holothuria cinerascens* in South Africa using ^1^H‐NMR and UPLC–QTOF–MS revealed distinct tissue‐specific and seasonal metabolic variations, with the body wall and gut/mesentery exhibiting high metabolite diversity. Findings highlight the bioactive potential of underexplored species and support future research into holothurian natural product discovery and sustainable aquaculture.

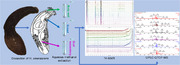

## 1 Introduction

Nutrition and natural drug research are interdisciplinary fields that employ diverse strategies to investigate the complex metabolic systems and physiological interactions within organisms [1, 2, 3]. Metabolomics, an emerging field within the ‘omics’ sciences, has gained prominence in natural drug discovery and development, clinical toxicology, pharmacology, biotechnology, nutrigenomics and functional genomics. The primary goal of metabolomics is the biochemical identification, classification and analysis of metabolites in biological samples, alongside studying how they change based on intrinsic and extrinsic factors [4, 5, 6].

Investigating the metabolome is vital for understanding physiological processes and responses in biological systems and discovering bioactive or medicinal compounds with potential applications in nutrition, aquaculture and biomedicine [1, 2, 7]. Naturally derived metabolites often exhibit significant health benefits with fewer side effects than synthetic pharmaceuticals, driving demand for these compounds in disease management and treatment [8, 9, 10]. However, the vast structural and chemical diversity of metabolites makes it difficult for any single method to assess. As a result, combinations of techniques are often used in a single investigation, with nuclear magnetic resonance (NMR) and liquid chromatography mass spectrometry (LCMS) being the most commonly employed procedures [3, 4, 6, 8].

Echinoderms, a group of marine invertebrates, are recognised as valuable sources of bioactive compounds. However, despite their potential, only about 6% of clinically tested natural products are derived from their extracts [10, 11, 12]. Sea cucumbers, soft‐bodied marine echinoderms, are consumed as a delicacy, referred to as ‘Beche‐de‐Mer’, ‘Gamat’ or ‘Trepang’, and hold significant medicinal value, particularly in Asian countries where they have been traditionally used to treat joint pain, arthritis, tendonitis and inflammation [13, 14, 15, 16, 17]. Sea cucumbers are rich in bioactive compounds, including saponins (triterpene glycosides), sulphated polysaccharides, mucopolysaccharides, glycosaminoglycans, peptides, fatty acids and polyphenols [12, 13, 18]. These compounds can exhibit a broad range of antimicrobial, anticoagulant, antihypertensive, cytotoxic, anticancer, antioxidant, anti‐inflammatory and wound healing properties, with bioactivities often linked to chemical or structural variations in their compounds [11, 12, 15, 17, 19]. As functional foods or nutraceuticals, sea cucumbers offer an appealing natural alternative for improving well‐being and preventing lifestyle diseases, aligning with a growing consumer preference for natural remedies over synthetic drugs [10]. Moreover, marine‐derived compounds are especially favoured due to their reduced risk of zoonotic diseases compared to terrestrial sources [10, 18].

Marine organisms, such as sea cucumbers, also produce unique secondary metabolites as adaptations to harsher marine environments, making them promising candidates for discovering novel bioactive compounds [9, 10, 11, 17, 18]. However, the metabolic composition of sea cucumbers can vary across species, tissue types and environmental conditions, reflecting their physiological state and nutritional value [20, 21, 22, 23, 24, 25]. This variability highlights the need for comprehensive metabolomic studies to explore these factors and their implications for human health, aquaculture and conservation. Despite their potential, research on the metabolic profiles of commercially overlooked, or ‘low‐value,’ sea cucumber species remains limited, with current studies focusing on only a few high‐value species. With over 1700 holothurian species worldwide [9], many neglected species may possess significant bioactive and nutritional potential. Expanding research efforts to include these species, particularly in developing regions, could provide valuable insights into their metabolic composition and environmental responses and promote sustainable cultivation and utilisation. Such initiatives are critical for addressing overexploitation, supporting conservation and fostering economic opportunities for developing nations.

Sea cucumbers are represented by at least 163 species in the waters surrounding South Africa alone [26]. Globally, members of the polyphyletic Aspidochirotida—comprising the Holothuriida, Stichopodida and Persiculida—are predominantly found in warm tropical and sub‐tropical regions. In South Africa, Holothuriida species are primarily found along the warmer Eastern coast, with limited occurrences in the colder Western coast regions [26]. Among the Holothuriida, the Holothuriidae family is the most diverse, encompassing 185 species across five genera, with *Holothuria* being the most speciose genus [19, 27, 28, 29]. *Holothuria cinerascens* is a benthic deposit feeder distributed circumglobally across South Africa and the Indian Ocean, primarily within low to middle latitudes [14, 28, 29, 30]. This species inhabits diverse habitats, including coral reefs, sandy environments and rocky shores, often in regions exposed to strong wave action [14, 28, 29, 30]. Averaging 16 cm in length, *H. cinerascens* can vary in colour, from rusty red–brown to dark purple or black [14, 27, 28, 29]. While primarily a deposit feeder, *H. cinerascens* can switch to non‐selective suspension feeding by extending its tentacles into the water column to capture food particles, such as microorganisms, plant debris, phytoplankton and other particulate matter [29, 30, 31]. The reproductive cycle of *H. cinerascens* follows the seasonal patterns typical of holothurians, with sexual reproduction occurring during the spring‐summer months and with gonads exhibiting various stages of development throughout the year [32, 33, 34]. In the Southern hemisphere, gonads are spent after January, with gametogenesis resuming by February. Notably, *H. cinerascens* may possess the ability to reproduce asexually, a process documented in several Aspidochirotida species such as *Holothuria leucospilota, Holothuria atra* and *Holothuria edulis* [35]; however, confirmation in *H. cinerascens* warrants further investigation. Additionally, *H. cinerascens* demonstrates a notable sensitivity to environmental stressors, as evidenced by its ability to eviscerate its internal organs as a defence mechanism against predation. This process is associated with metabolic alterations, likely involving saponins–bioactive compounds that play crucial roles in holothurian defence. Studies on *Holothuria* sp. have suggested that oxidised and sulphated saponins are largely involved in holothurian defence mechanisms, particularly in species lacking Cuvierian tubules [11, 19, 20]. This underscores the potential of *H. cinerascens* as a source of bioactive metabolites, offering promising implications for human health, biomedical applications and aquaculture integration.

Despite its potential ecological and economic significance, the metabolic composition of *H. cinerascens* remains poorly understood, particularly in populations from the coasts of Southern Africa. To address this knowledge gap, this study conducted an untargeted metabolomic analysis on *H. cinerascens* specimens collected from KwaZulu‐Natal, South Africa. Using Proton NMR and full‐scan ultra performance liquid chromatography quadruple time‐of‐flight mass spectrometry (UPLC–QTOF–MS), aiming to investigate the metabolite profiles of different body tissues from *H. cinerascens*—specifically the body wall, gonad and gut/mesentery—across summer and winter. By doing so, this research seeks to enhance the global understanding of holothurian metabolomics and identify seasonal metabolic variations and the potential for novel bioactive compounds within *H. cinerascens*. These findings provide valuable insights into the metabolic composition of *H. cinerascens*, highlighting its potential as a source of economically and pharmaceutically valuable natural bioactive compounds and supporting its feasibility for integration into sustainable aquaculture systems. By exploring these aspects of holothurian biology, this study contributes to advancing holothurian research, promoting sustainable resource utilisation and highlighting opportunities for research development in resource‐limited regions.

## 2 Methods and Materials

### 2.1 Ethical Statement

Sea cucumbers are lower order marine invertebrates that at the time of study did not require ethics clearance under the University of KwaZulu‐Natal Animal Research Ethics Committee (AREC) (https://research.ukzn.ac.za/research‐office/ethics‐overview/animal‐ethics/). Although ethics clearance was not required, collection and transportation were carried out per the Department of Environment, Forestry and Fisheries (DFFE) and the University of KwaZulu‐Natal recommendations.

### 2.2 Sample Collection and Preparation

*H. cinerascens* (Brandt, 1835) specimens were collected during low spring tide from the rocky intertidal region at Park Rynie, KwaZulu‐Natal, South Africa (30° 19′ 246 S; 30° 44′ E), in August 2021 (Winter) and January 2022 (Summer) periods. Ten specimens were collected per season, frozen, washed clean and dissected into their body wall, gonads and mesentery/gut tissue. The final sample compilation consisted of three biological replicates (indicated as HC 1, 2 and 3) per season (S or W), each comprising technical replicates assessing the body wall (a), gonad (b) and gut/mesentery tissues (c). They were then frozen in liquid nitrogen, freeze‐dried, ground into a fine powder and stored at −80°C until analysis.

### 2.3 NMR Analysis

^1^H‐NMR analysis was performed in triplicate for each tissue type (body wall, gonad and gut/mesentery) from summer and winter samples at the Council for Scientific and Industrial Research (CSIR), Pretoria. This amounted to three biological replicates of each tissue type per season. The 0.01% TSP, D_2_O–KH_2_PO_4_ buffer was prepared by adding 1.2 g of potassium dihydrogen phosphate (KH_2_PO_4_) and 10 mg of the standard compound 3—(trimethylsilyl)propionic acid—d4 (TSP) sodium to 100 mL of deuterium oxide (D_2_O). The solution was mixed until all the particles were dissolved, and the pH was adjusted to 6.0 using 1 M NaOH.

To prepare the samples for analysis, 50 mg of the dried powder was weighed and placed into a 2 mL Eppendorf tube. Equal volumes, 600 µL, of deuterated methanol (CH_3_OH‐d_4_) and a 0.01% TSP, D_2_O–KH_2_PO_4_ buffer were added to the sample. The sample was placed in a sonicator for 15 min, followed by centrifugation at 12 000 rpm (10 625 × *g*) for 15 min. The supernatant was carefully collected and transferred to an NMR tube. Spectra were acquired by performing 32 scans using a 28‐shim Varian VNMRS (DDR‐1) PremiumShield system operating at a nominal proton frequency of 600.13 MHz. The instrument was configured with a dedicated 5 mm H{CN} triple‐resonance probe (room temperature, indirect detection), integrated with a pulsed field gradient generator, an Agilent 7510‐AS 12‐position autosampler, and a system computer running VNMR 4.2A software. All spectra were collected under automated conditions for locking, shimming and acquisition, using standard 1D PROTON parameters, which included a spectral width of 14 ppm, a 45° pulse angle, no recycle delay, and a fixed temperature of 30°C.

### 2.4 Multivariate Data Analysis

MestReNova (v14.2.2) was used to pre‐process the raw spectral data by referencing the TSP standard, normalising and performing a baseline correction on the spectral data. The spectral intensities were then reduced into integrated regions at 0.04 ppm width bins between 0.04 and 10 ppm.

The transformed data were then exported as Microsoft Excel CSV for multivariate analysis using the Soft Independent Modelling of Class Analogy (SIMCA) software V17.0.1 (Umetrics, Sweden). The residual water peak (4.6–5.0 ppm) and methanol peaks (3.28–3.36 ppm) were excluded from the dataset for final analysis. The data were first analysed using an unsupervised principal component analysis (PCA) to generate scatter plots to reduce the dimensionality of the data and identify trend clusters and potential data outliers. This was followed by a supervised orthogonal partial least squares discriminant analysis (OPLS‐DA). Score and contribution plots were used to determine the sources of variation between the body tissues and seasons of *H. cinerascens*. The OPLS‐DA models were validated using the permutation test carried out with 100 permutations. Compound annotation was then done using the chemical shift values generated from the contribution plots and Mnova in conjunction with online compound databases, such as Chenomx (v9.02) and published literature.

### 2.5 Full‐Scan Ultra‐Performance Liquid Chromatography Quadruple Time‐of‐Flight Mass Spectrometry

Metabolites were extracted from biological replicates of each tissue type (body wall, gonad and gut/mesentery) from summer and winter samples of *H. cinerascens*. Approximately 45 mg of freeze‐dried tissues were combined with 2 mL of cold 70% HPLC‐grade methanol. The mixture underwent sonication and vortexing, followed by centrifugation. The supernatant was carefully collected and filtered through a 0.2‐micron Pall Acrodisc GHB filter (13 mm; 0.2 µm) and a glass syringe with a Teflon plunger head for UPLC analysis.

The initial UPLC–QTOF–MS analysis was performed using a Waters Classic UPLC system coupled with a Waters SYNAPT G1 HDMS mass spectrometer (Waters Corporation, Milford, MA, USA). However, due to mass spectral signal saturation and co‐elution issues, the analysis was repeated on a Waters Acquity Premier UPLC system linked to a Waters SYNAPT XS HDMS mass spectrometer (Waters Corporation, Milford, MA, USA).

Metabolite separation was achieved using a Waters T3 C18 column (150 mm × 2.1 mm, 1.8 µm) maintained at 60°C, with a 0.4 mL/min flow rate. The mobile phase consisted of Solvent A (water with 10 mM formic acid) and Solvent B (Acetonitrile with 10 mM formic acid). The elution gradient was programmed to start at 100% Solvent A for 1 min, transitioning linearly to 1% Solvent A at 16 min, with a total runtime of 20 min.

Mass spectrometric analysis was performed using both positive and negative electrospray ionisation modes (ESIPos and ESINeg) to acquire Centroid data under the following operating conditions: capillary voltage, 0.6 kV; sample cone voltage, 30 V; source temperature, 120°C; desolvation temperature, 450°C; desolvation gas, nitrogen (N_2_) with a flow rate of 600 L/h. LockMass correction was applied by sampling the standard leucine enkephalin (100 pg/µL) every 20 s.

The data generated from the UPLC–QTOF–MS analysis was processed with a mass accuracy maintained below 1 MDa using the MassLynx 4.2 software (SCN 1028) built into the SYNAPT XS system. MassLynx 4.2 was also used to analyse elemental composition using the embedded elemental composition software. Predefined element ranges were set for carbon (C) (1–80), hydrogen (H) (1–200), oxygen (O) (0–35) and sulphur (S) (0–2) to ensure a comprehensive evaluation of possible elemental compositions related to triterpene glycosides, while excluding atypical elements related to sea cucumbers, such as chlorine (Cl), fluorine (F), nitrogen (N) and phosphorous (P). Only monoisotopic mass data were used, with both odd and even electron states considered with a mass tolerance of 3 MDa. Metabolite identification was performed by comparing the mass spectral data with reference information from online databases, including ChemSpider, PubChem, the Dictionary for Marine Natural Products, and the NIST 2014 Mass Spectral Library and by consulting relevant literature.

## 3 Results

**FIGURE 1:**
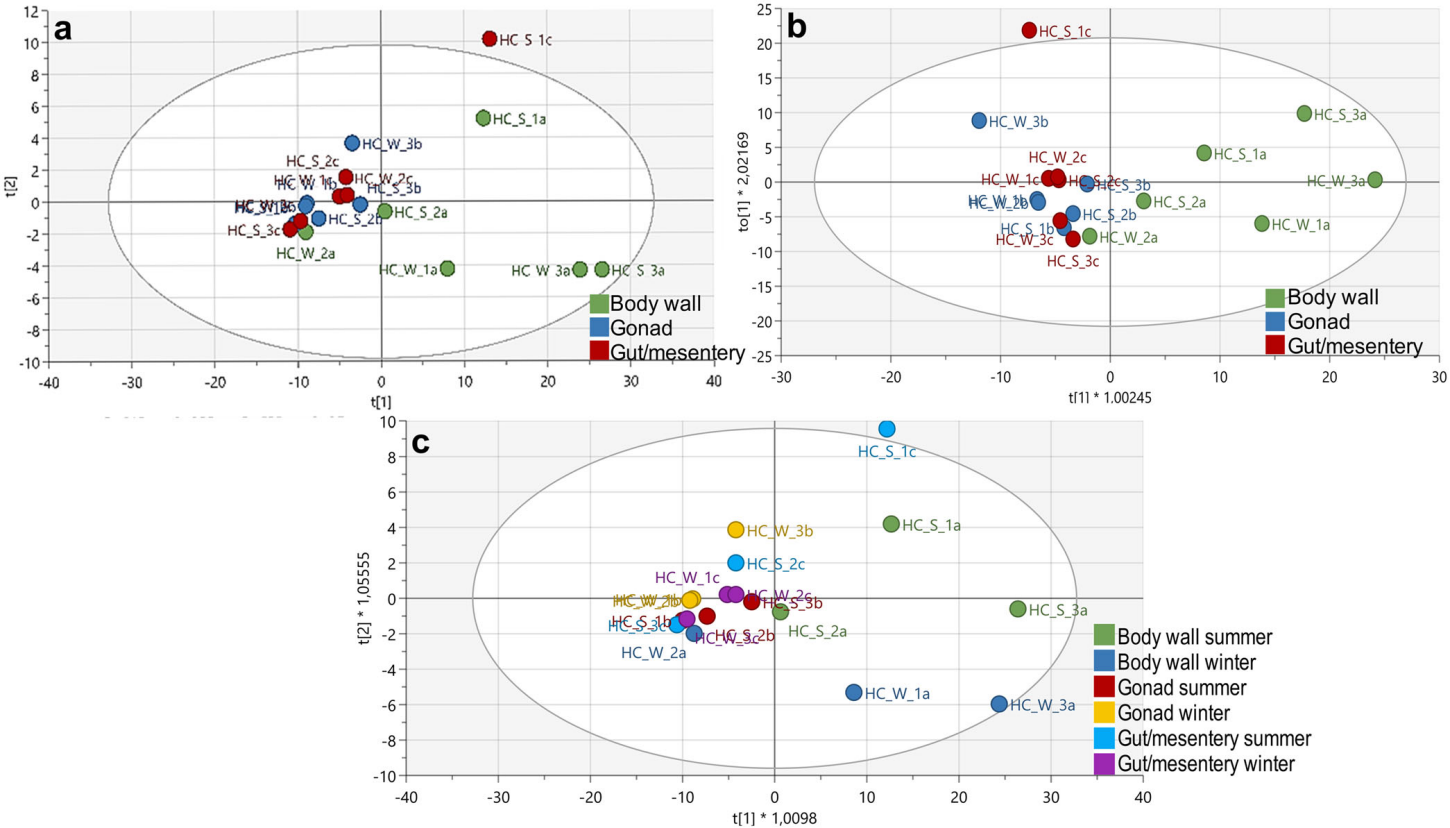
A representation of the PCA‐X results (a) and OPLS‐DA score plots from the ^1^H‐NMR spectral data showing the separation of body tissues (b) in *Holothuria cinerascens* over summer and winter (c).

The ^1^H‐NMR dataset was analysed using SIMCA v17.0.1 to construct PCA‐X and OPLS‐DA plots to compare the chemical profiles of various body tissues from *H. cinerascens* during summer and winter. PCA, an unsupervised multivariate analysis technique that identifies metabolic variations across groups [36, 39], was initially performed to ensure an unbiased comparison of the samples. The results, illustrated in Figure 1a, revealed a principal cluster comprising gonad and gut/mesentery body tissues. In contrast, the body wall exhibited significant differentiation, both between the tissues and within the tissue group, separating distinctly from the remaining body tissues. The close clustering of the gonad and gut/mesentery tissues implied a higher degree of metabolic similarity between these tissues than the body wall. Notably, one summer gut/mesentery sample (‘HC_S_1c’) displayed significant differentiation from the other samples within its class; however, it was not identified as an outlier according to the DModX line plot. Additionally, one winter body wall sample (‘HC_W_2a’) demonstrated greater similarity to the other tissues and was positioned within the main cluster. The PCA‐X results showed a high level of predictability and goodness‐of‐fit, as indicated by an *R* ^2^ *X*(cum) of 0.946 and *Q* ^2^(cum) of 0.842.

Subsequently, the OPLS‐DA score plot (Figure 1b), a supervised multivariate pattern recognition analysis designed to discriminate between samples based on targeted class allocation, was conducted. The OPLS‐DA results further accentuated the PCA‐X separation of body wall tissues from the other body tissues. The gonad and gut/mesentery tissues remained grouped as a single cluster, with ‘HC_S_1c’ now positioned on the same side of the *y*‐axis as the main clustering of the gut/mesentery samples. Notably, despite ‘HC_S_1c’ appearing outside the 95% confidence circle, it did not qualify as an outlier according to the DModX line plot. The OPLS‐DA model featured an *R* ^2^ *X*(cum) of 0.898, a goodness of fit (*R* ^2^ *Y*) of 0.348 and a low predictability of *Q* ^2^(cum) = 0.194. To validate the model, a permutation plot involving 100 permutations was executed and had the intercepts as *R* ^2^ = (0.0; 0.117) and *Q* ^2^ = (0.0; −0.249) (Figure S1). A *Q* ^2^(cum) value greater than 0.5 suggests a good predictive power within the model. The lower values observed here suggest that there may be too little deviation between the sample groups, potentially limiting the model's predictive accuracy.

Furthermore, there appeared to be no distinct seasonal demarcation across all the body tissues (Figure 1c). The seasonal model had featured an *R* ^2^ *X*(cum) of 0.971, a goodness of fit (*R* ^2^ *Y*) of 0.524 and a low predictability of *Q* ^2^(cum) = 0.00856. The results suggest that variations in body tissue may play a more substantial role in metabolic diversity than seasonal changes. Additionally, the body wall of *H. cinerascens* appears to exhibit the most notable metabolic differentiation in contrast to the gonad and gut/mesentery tissues, which display a higher degree of metabolic similarity.

To determine the metabolic differences between the various body tissues of *H. cinerascens*, NMR spectral data from each body tissue were compared by creating spectral stacks (Figure S2). Despite their close clustering, the results identified pronounced metabolic similarities between the gonad and gut/mesentery tissues, while revealing distinct metabolic variations that set the body wall apart from these two tissues. Notably, the body wall samples exhibited less refined spectral peak patterns than the gonad and gut/mesentery tissues.

In the sugar–aliphatic region (0.8–6.0 ppm), the body wall exhibited a series of high‐intensity peaks between 0.8 and 1.6 ppm, likely corresponding to straight‐chain alkyl or aliphatic compounds such as butyrate (*δ* ∼1.1), isovalerate (*δ* ∼0.9) and valerate (*δ* ∼0.8, 1.3, 1.5), as well as aliphatic amino acids, distinguishing it from the other two tissues. A doublet around 1.1 ppm was present in the body wall and shared by a few gonad and gut/mesentery samples. At 1.5 ppm, a doublet was evident in the gonad and gut/mesentery tissue, with higher abundance in the winter gonads, contrasting with a singlet observed in the body wall. Between 1.7 and 2.5 ppm, the body wall displayed several low‐resolution peaks and a multiplet or sloped peak at approximately 2.4 ppm. In contrast, the gonad and gut/mesentery tissues exhibited a range of multiplets spanning this region, although these appeared less defined in the winter gut/mesentery spectra. In the 3.0–4.0 ppm region, distinct spectral variations were observed among the three tissues, likely corresponding to sugars, such as galactose, glucose and xylose, which form major components of triterpene glycosides, as well as amino acids like serine and taurine. The body wall displayed the highest metabolite concentrations, whereas the gonad and gut/mesentery samples showed greater spectral similarities. However, peaks in the winter gut/mesentery tissues appeared less defined. Notably, the body wall featured a sloped peak around 4.2 ppm, absent in the other tissues, and two doublets between 4.5 and 4.6 ppm, observed only in one gonad sample, ‘HC_S_2b’. In the high‐sugar aromatic region (5.5–9.0 ppm), the body wall displayed two doublets around 5.4 ppm, which appeared as a sloped peak in the gonad and gut/mesentery tissues, likely corresponding to sugar protons. The gut/mesentery tissues contained a greater abundance of singlets between 5.2 and 5.4 ppm compared to the body wall or gonad samples. Notably, the gonad and summer gut/mesentery samples shared a doublet around 6.15 ppm, with the summer gonads showing an additional signal at 5.95 ppm. Between 8.1 and 8.6 ppm, the body wall exhibited few spectral peaks aside from a minor signal around 8.55 ppm, whereas the gonad and summer gut/mesentery tissues displayed several aromatic peaks. Overall, these findings highlight subtle seasonal variability within tissue types but indicate limited seasonal variation across all samples. The body wall displayed clear metabolic distinction from the gonad and gut/mesentery tissues, which were more similar to each other.

### 3.1 Tissue X Tissue Pairwise Comparisons

**FIGURE 2:**
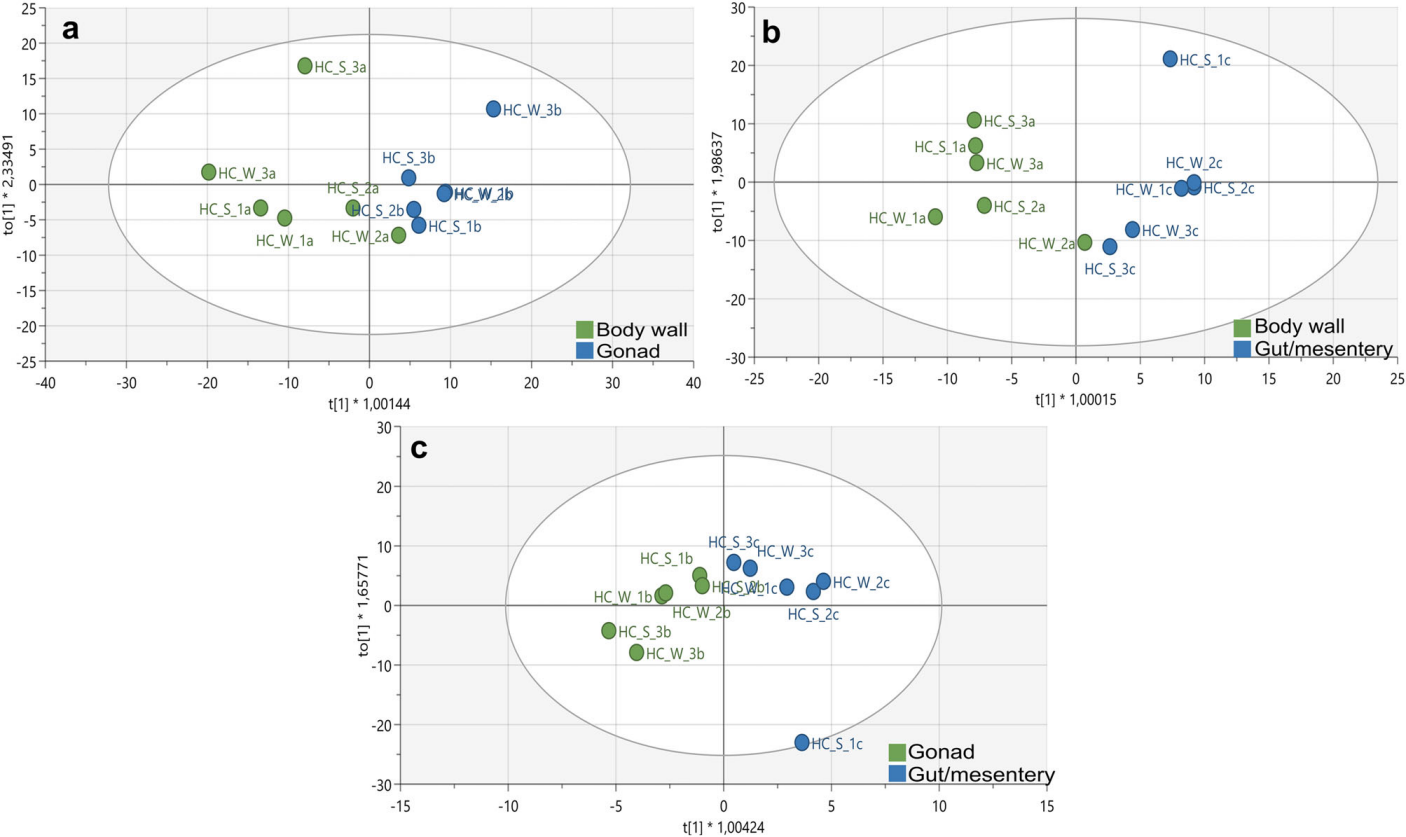
OPLS‐DA score plots from the ^1^H‐NMR spectral data showing the body wall versus gonad (a), body wall versus gut/mesentery (b) and gonad versus gut/mesentery (c) body tissue comparison in *Holothuria cinerascens*.

Due to the low predictability and limited differentiation observed in the overall tissue comparison (Figure 1; *Q* ^2^(cum) = 0.194), pairwise OPLS‐DA analyses were conducted for the body wall versus gonad (Figure 2a), body wall versus gut/mesentery (Figure 2b) and gonad versus gut/mesentery (Figure 2c). These targeted comparisons aimed to more precisely visualise metabolic separations among the tissues and to identify any significantly differentiated pairings. The pairwise results support and expand on the trends observed in the ^1^H‐NMR spectral stack (Figure S2).

Each model demonstrated robustness with moderate yet reliable predictability, with the body wall versus gonad having an *R* ^2^ *X*(cum) = 0.912, *R* ^2^ *Y*(cum) = 0.665 and *Q* ^2^(cum) = 0.4, the body wall versus gut/mesentery having an *R* ^2^ *X*(cum) = 0.978, *R* ^2^ *Y*(cum) = 0.831 and *Q* ^2^(cum) = 0.596 and the gonad versus gut/mesentery having an *R* ^2^ *X*(cum) = 0.932, *R* ^2^ *Y*(cum) = 0.775 and *Q* ^2^(cum) values = 0.434.

Among these comparisons, the body wall vs gut/mesentery exhibited the most significant metabolic separation, indicating more pronounced biochemical differentiation between these two tissues. All models were validated using permutation tests with 100 permutations to confirm their reliability (Figure S3).

**FIGURE 3:**
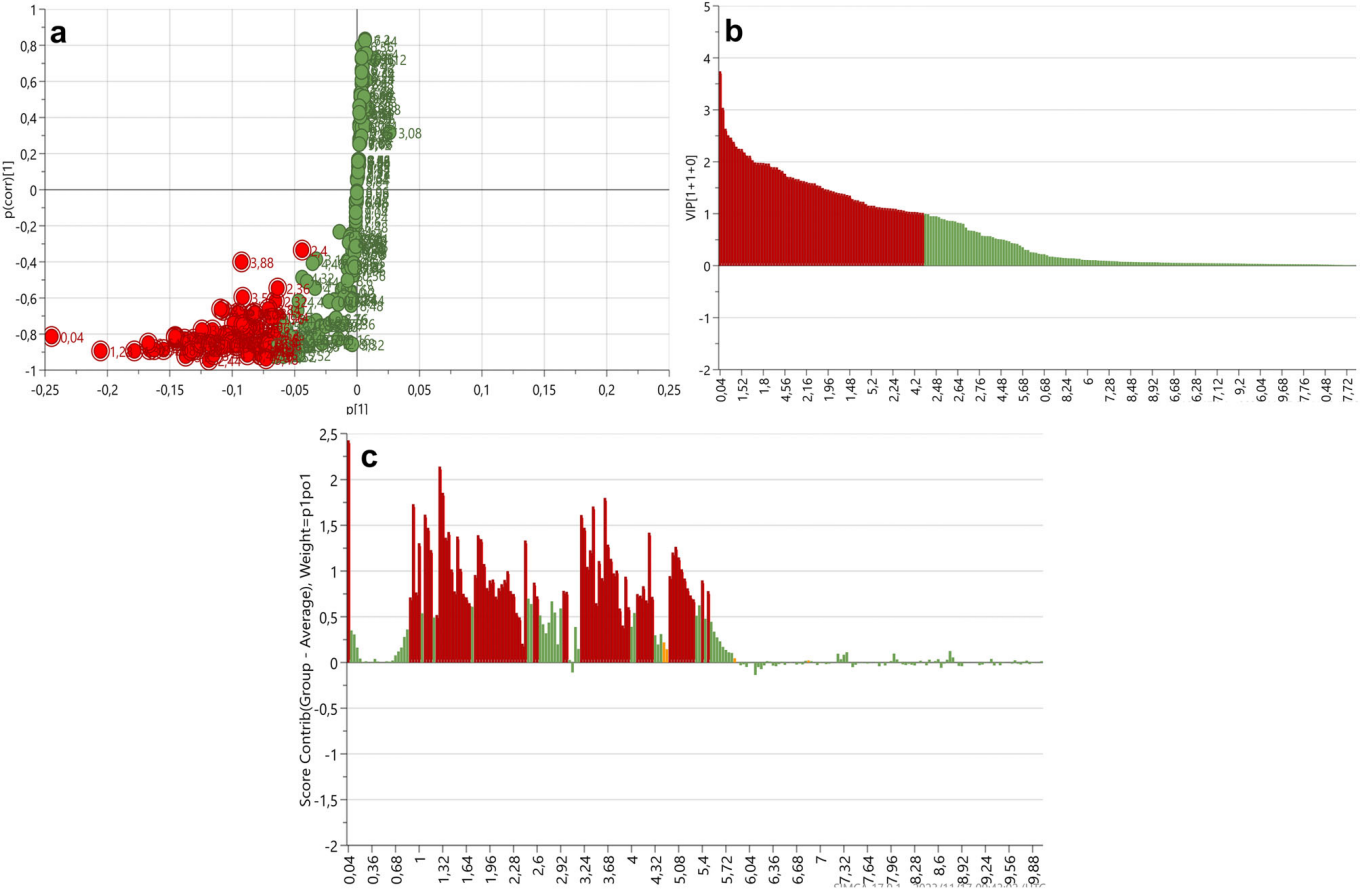
S‐plot (a), VIP score plot (b) and contribution loadings plot (c) showing ^1^H‐NMR spectral regions spectral regions contributing to the clustering and separation of the body wall versus gonadal tissues from *Holothuria cinerascens*. Bars above the *X*‐axis (c) showing regions positively associated with the body wall tissue from *H. cinerascens*.

**FIGURE 4:**
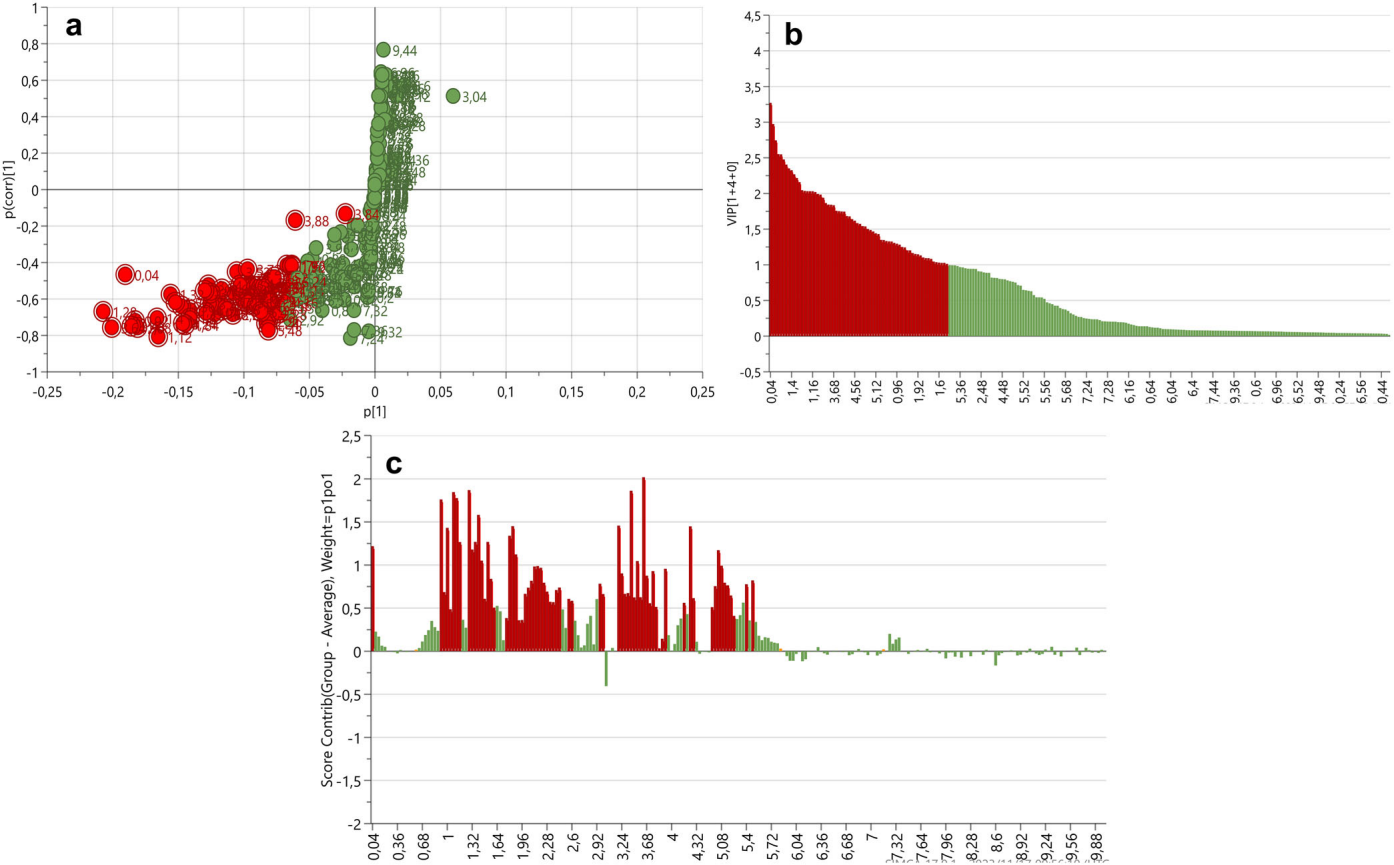
S‐plot (a), VIP score plot (b) and contribution loadings plot (c) showing ^1^H‐NMR spectral regions contributing to the clustering and separation of the body wall versus gut/mesentery tissues from *Holothuria cinerascens*. Bars above the *X*‐axis (c) showing regions positively associated with the body wall tissue.

**FIGURE 5:**
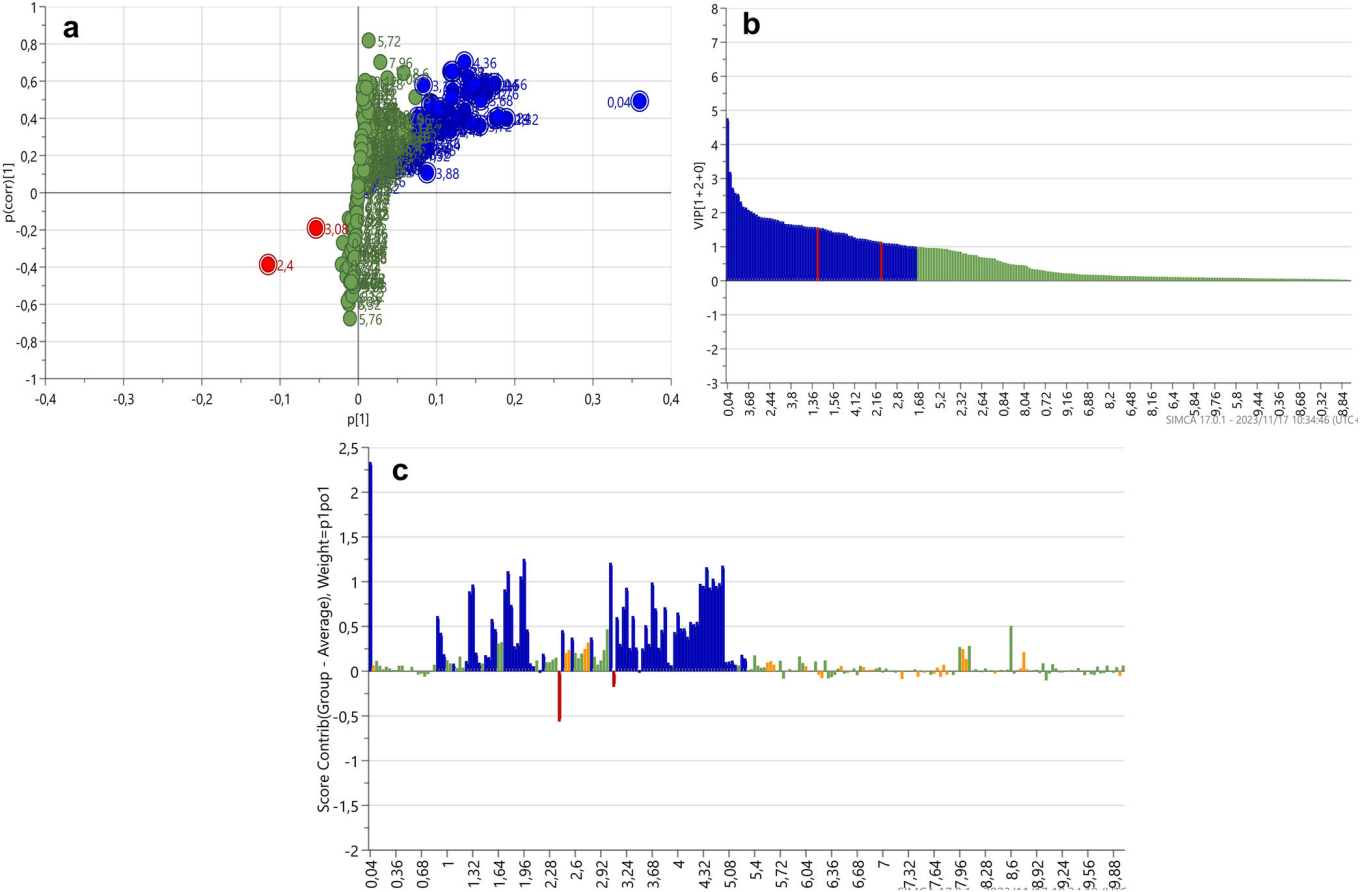
S‐plot (a), VIP score plot (b) and contribution loadings plot (c) showing ^1^H‐NMR spectral regions contributing to the clustering and separation of the gonadal versus gut/mesentery tissues from *Holothuria cinerascens*. Bars above the *X*‐axis (c) showing regions positively associated with the gut/mesentery tissue.

S‐plots and VIP scores plots were generated for each tissue comparison to determine the spectral regions influencing the separation between the body wall versus gonad (Figure 3a,b), body wall versus gut/mesentery (Figure 4a,b) and gonad versus gut/mesentery (Figure 5a,b). The influential regions were identified from both ends of the S‐plots and VIP scores ≥ 1.0. Contribution loadings plots were generated for each tissue comparison to visualise the influential spectral regions.

The ^1^H NMR bins obtained from the contribution loading plots (Figures 3c, 4c and 5c) representing spectral regions of importance contributing to the clustering and metabolic separation of the tissues were annotated using online database comparisons (Chenomx and HMDB) and relevant literature. Possible metabolite annotations were evaluated and reported in Tables S1 and S2.

The contribution loadings plot comparing the body wall and gonadal tissue (Figure 3c) identified the regions positively associated with the body wall (and negatively associated with the gonadal tissue) at *δ* 0.04, 0.92, 1, 1.08,1.12, 1.16, 1.28, 1.32, 1.36, 1.4, 1.44, 1.52, 1.56, 1.76, 1.8, 1.84, 1.88, 2.16, 2.44, 3.2, 3.24, 3.4, 3.44, 3.48, 3.56, 3.64, 3.68, 3.72, 3.76, 3.92, 4.24, 4.52, 4.56, 5.04, 5.08, and to a lesser extent 0.88, 0.96, 1.24, 1.48, 1.6, 1.64, 1.68, 1.92, 1.96, 2, 2.04, 2.08, 2.12, 2.2, 2.24, 2.28, 2.32, 2.36, 2.4, 2.56, 2.6, 2.96, 3, 3.52, 3.6, 3.8, 3.84, 3.88, 3.96, 4.08, 4.12, 4.16, 4.2, 4.28, 5.12, 5.16, 5.2, 5.24, 5.28, 5.4 and 5.48 ppm. On the basis of the contribution values and metabolite annotations (Table S1 and S2), several amino acids were more abundant in the body wall tissue, differentiating it from the gonadal tissue. These included alanine (*δ* ∼1.48, 3.76 ppm), glutamate (*δ* ∼2.04, 2.12, 2.36 ppm), glycine (*δ* ∼3.56 ppm), taurine (*δ* ∼3.24, 3.44 ppm) and threonine (*δ* ∼1.32, 3.6 and 4.2 ppm), suggesting structural protein metabolism in the body wall. Organic acids, including butyrate (*δ* ∼0.88, 1.52 and 2.16 ppm), isobutyrate (*δ* ∼1.08, 2.4 ppm), malonate (*δ* ∼3.2 ppm), methylmalonate (*δ* ∼1.24, 3.2 ppm), valerate (*δ* ∼0.88, 1.32, 1.52 and 2.16 ppm) and isovalerate (*δ* ∼0.88, 1.92 and 2.04 ppm), were also identified along with dimethyl sulfone (*δ* ∼3.2 ppm), glucose (*δ* ∼3.24, 3.4, 3.44, 3.48, 3.52, 3.72, 3.8, 3.88 and 5.2 ppm) and xylose (*δ* ∼3.2, 3.4, 3.44, 3.48, 3.6, 3.64, 3.88, 4.52 and 5.2 ppm). In contrast, the gonadal tissue showed higher levels of betaine (*δ* ∼3.24, 3.84 ppm), pyruvate (*δ* ∼2.36 ppm) and lysine (*δ* ∼1.44, 1.52, 1.88, 1.92, 3.00 and 3.76 ppm).

The contribution loadings plot comparing the body wall and gut/mesentery tissues (Figure 4c) identified the regions positively associated with the body wall at *δ* 0.04, 0.92, 1.00, 1.08, 1.12, 1.16, 1.28, 1.32, 1.36, 1.40, 1.44, 1.52, 1.56, 1.80, 1.84, 1.88, 2.08, 2.12, 2.16, 2.20, 2.40, 2.44, 3.20, 3.24, 3.40, 3.48, 3.52, 3.56, 3.64, 3.68, 3.72, 3.76, 3.92, 4.24, 4.56, 5.04, 5.08, 5.12, and to a lesser extent, 0.96, 1.04, 1.48, 1.60, 1.76, 1.92, 1.96, 2.00, 2.04, 2.24, 2.28, 2.32, 2.36, 2.56, 2.60, 2.96, 3.00, 3.44, 3.60, 3.80, 3.84, 3.88, 4.16, 4.28, 4.52, 5.16, 5.20, 5.24, 5.40 and 5.48 ppm. On the basis of Chenomx and literature‐based annotations (Tables S1 and S2), metabolites likely associated with these regions include the amino acids: alanine (*δ* ∼1.48, 3.76 ppm), glutamate (*δ* ∼2.04, 2.12, 2.36, 2.40 and 3.76 ppm), glycine (*δ* ∼3.56 ppm), threonine (*δ* ∼1.32, 3.60 ppm) and taurine (*δ* ∼3.24, 3.40 ppm). In addition, short‐chain fatty acids and carboxylic acids—including butyrate (*δ* ∼1.52, 2.16 ppm), isobutyrate (*δ* ∼1.04, 2.40 ppm), valerate (*δ* ∼1.32, 1.52 and 2.16 ppm), isovalerate (*δ* ∼0.92, 1.92 and 2.04 ppm), malonate (*δ* ∼3.20 ppm) and methylmalonate (*δ* ∼3.20 ppm)—were more abundant in the body wall tissue.

The contribution loadings plot comparing the gonad and gut/mesentery tissues (Figure 5c) identified the regions at *δ* 0.04, 0.88, 0.92, 1.28, 1.32, 1.56, 1.72, 1.76, 1.80, 1.92, 1.96, 2.44, 3.04, 3.2, 3.24, 3.44, 3.6, 3.68, 3.72, 3.8, 3.84, 3.96, 4.0, 4.04, 4.24, 4.28, 4.32, 4.36, 4.4, 4.44, 4.48, 4.52, 4.56, and to a lesser extent 0.96, 1.08, 1.24, 1.36, 1.4, 1.48, 1.52, 1.6, 1.84, 1.88, 2.00, 2.04, 2.08, 2.16, 2.20, 2.56, 2.80, 3.12, 3.16, 3.40, 3.48, 3.52, 3.56, 3.64, 3.76, 3.88, 3.92, 4.08, 4.12, 4.16, 4.20, 5.04, 5.08, 5.12, 5.16, 5.24 and 5.28 ppm as positively associated with the gut/mesentery samples. Negatively associated regions were identified at *δ* 2.40 and 3.08 ppm. On the basis of tentative annotations (Tables S1 and S2), the gut/mesentery tissue showed higher levels of sugars and polyols, including glucose (*δ* ∼3.24, 3.40, 3.44, 3.52, 3.72, 3.76, 3.80, 3.88 and 5.24 ppm), xylose (*δ* ∼3.20, 3.44, 3.48, 3.60, 3.68, 3.88 and 4.52 ppm), galactose (*δ* ∼3.48, 3.64, 3.68, 3.72, 3.76, 3.80, 3.84, 3.92, 3.96, 4.00, 4.08, 4.56 and 5.28 ppm) and glycerol (*δ* ∼3.52, 3.60 and 3.72 ppm). The gut/mesentery tissue also displayed higher levels of amino acids, such as glycine (*δ* ∼3.56 ppm), lysine (*δ* ∼1.40, 1.48, 1.72, 1.88, 1.92 and 3.76 ppm), serine (*δ* ∼3.84, 3.92 and 3.96 ppm), glutamate (*δ* ∼2.04, 2.08 and 3.76 ppm) and taurine (*δ* ∼3.24, 3.44 ppm), along with organic acids, including butyrate (*δ* ∼0.88, 1.52 and 2.16 ppm), methylmalonate (*δ* ∼1.24, 3.16 ppm) and ascorbic acid (*δ* ∼3.72, 3.76, 4.04 and 4.52 ppm). In contrast, the gonadal tissue had a higher abundance of osmolytes and energy metabolites, betaine (*δ* ∼3.24, 3.84 ppm), malonate (*δ* ∼3.12 ppm) and pyruvate (*δ* ∼2.4 ppm). These differences reflect metabolic specialisation between these two tissues, indicating distinct roles in carbohydrate and amino acid metabolism, as well as osmoregulatory activities.

Compound annotation was conducted comparing the body wall, gonadal and gut/mesentery tissues of *H. cinerascens* to elucidate metabolic variability between tissues. Overall, the comparisons revealed distinct differences in metabolite composition and abundance among the tissues, providing insights into their metabolic activities and potential functional roles within *H. cinerascens*. The body wall and gut/mesentery tissues exhibited greater metabolite diversity and abundance than the gonadal tissue (Table S2). Tentative quantification using Chenomx indicated elevated levels of metabolites associated with osmoregulation, protein synthesis and energy metabolism. In the body wall, the presence of several amino acids and sugar monosaccharides suggests involvement in peptide metabolism or the biosynthesis of saponin‐type compounds, which are particularly abundant in holothurian body walls. Relative comparisons also revealed a higher abundance of amino acids, short‐chain fatty acids and dicarboxylic acids in the body wall, underscoring its metabolic versatility. In contrast, the gut/mesentery tissue contained higher levels of energy‐related compounds, such as glucose and galactose, likely reflecting food metabolism and microbial activity. Although the gonad exhibited the lowest overall metabolite abundance, it showed the highest levels of betaine, an osmoregulatory compound and pyruvate, a key intermediate in energy metabolism.

## 4 Seasonal Comparisons Within Body Tissues

### 4.1 Body Wall

**FIGURE 6:**
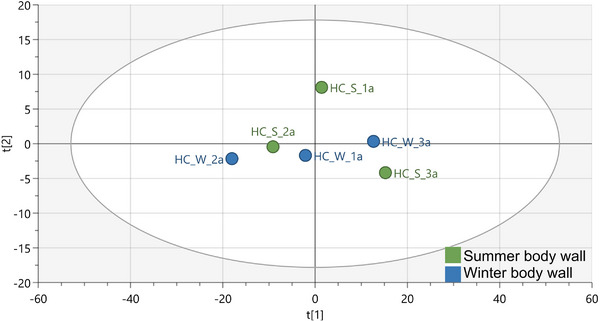
Representation of the PCA‐X score plot results from the ^1^H‐NMR spectral data showing the body wall tissue comparison between *Holothuria cinerascens* over summer and winter.

The PCA‐X results (Figure 6) comparing the body wall of *H. cinerascens* across summer and winter did not indicate significant overall seasonal variation. The robustness of the model was supported by high values of *R* ^2^ *X* = 0.931 and *Q* ^2^(cum) = 0.74. Notably, winter samples showed limited vertical separation, whereas summer samples exhibited both vertical and horizontal dispersion. This suggests that although overall variability was low, the summer samples display greater metabolic variability, potentially influenced by seasonal changes in internal and external factors.

Notably, *H. cinerascens* has been observed to undergo evisceration under stress conditions as a defence mechanism. During this process, specific metabolites are released, which trigger the expulsion of internal body tissues. *H. cinerascens* may also have the potential to undergo asexual reproduction during winter in the southern hemisphere, a process seen in several Aspidochirotida species, such as *H. leucospilota*, that involves metabolic changes that alter the mutable collagenous tissue within the body wall to facilitate transverse fission, though this warrants further investigation [41]. Additionally, the availability of food sources can significantly impact the metabolic profile of sea cucumbers. Therefore, it is plausible that the observed metabolic variability, or lack thereof, may be linked to the species’ access to a stable food supply or other environmental factors that influence their metabolic processes.

NMR spectral stacks (Figure S4) were generated to visualise the peak distribution patterns across the body wall samples of *H. cinerascens* collected during summer and winter. These results align with the PCA‐X findings, indicating minimal metabolite differentiation between the two seasons, with variations primarily reflected in peak intensities.

The spectral profiles of summer and winter body wall samples displayed substantial similarities across the aliphatic (0.5–3.0 ppm), sugar (3.0–6.0 ppm) and aromatic (6.0–9.0 ppm) regions. The winter samples ‘HC_W_1a’ and ‘HC_W_3a’ exhibited slightly higher metabolite intensities than the summer samples, whereas ‘HC_W_2a’ displayed lower peak intensities (Figure S4a), consistent with its wider dispersion in the PCA‐X plot (Figure 6).

In the sugar–aliphatic region, *H. cinerascens* exhibited distinct spectral features, characterised by a series of doublets and a singlet between 0.8 and 1.6 ppm. The 1.7–2.5 ppm region displayed a collection of low‐resolution peaks and a multiplet or sloped peak at approximately 2.4 ppm, likely corresponding to amino acids such as glutamine and glutamate or to sugar alcohols. Notably, numerous peaks of varying intensities were observed between 3.5 and 3.9 ppm, indicative of sugar‐containing metabolites and possibly triterpene glycosides. A doublet or shouldered peak was also observed around 4.25 ppm, with the winter samples ‘HC_W_1a’ and ‘HC_W_3a’ showing a more pronounced dual peak pattern between 4.1 and 4.2 ppm. The body wall spectra also featured a singlet around 4.4 ppm, two doublets between 4.5 and 4.6 ppm, and a minor doublet around 4.65 ppm. In the aromatic region, the body wall spectra displayed two singlets or minor doublets between 5.3 and 5.5 ppm, likely corresponding to sugars such as glucose or xylose, and a singlet at approximately 6.8 ppm that appeared more consistent in the summer samples. A series of minor doublets or singlets between 8.0 and 8.8 ppm indicated aromatic protons, though these were less pronounced in ‘HC_S_2a’ and ‘HC_W_2a’.

Overall, the results indicate limited metabolic differentiation between the summer and winter body wall tissues, with minor concentration differences observed within and between the seasonal replicates. Chenomx‐based evaluations showed slight metabolite deviations, which may account for the broader dispersion of summer samples compared to winter. These differences likely reflect natural physiological or environmental variations. Sea cucumbers are known to be rich in triterpene glycosides, especially holothurin‐type compounds, which typically exhibit characteristic peaks in the sugar–aliphatic spectral region. The spectral features observed in the 3.0–5.50 ppm region (Figure S4) may correspond to these compounds, which are abundant in the *Holothuria* genus [19, 20]. Although no strong seasonal separation was observed in the PCA‐X, several metabolites displayed slight seasonal variation (Table S2). Noteworthy compounds included alanine, glutamate, glycine, lysine, d‐glucose, d‐xylose, galactose, isobutyrate, glycerol, ascorbic acid and pyruvate, which were slightly higher in summer and can be seen in Figure S4b, reflecting increased energy metabolism and potential saponin biosynthesis in warmer months. In contrast, winter samples showed increases in myo‐inositol, malonate, methylmalonate, arginine, taurine, dimethyl sulfone, valerate and butyrate, which may be associated with enhanced osmoprotection and metabolic adaptation to cooler conditions.

### 4.2 Gonad

**FIGURE 7:**
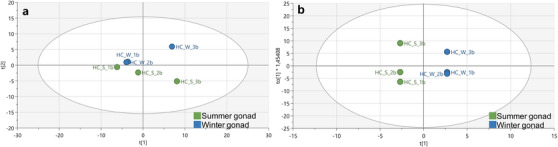
A representation of the PCA‐X results (a) and OPLS‐DA score plot (b) from the ^1^H‐NMR spectral data showing the seasonal separation of gonadal tissue samples from *Holothuria cinerascens*.

To assess the extent of metabolite variation between gonadal tissue samples from *H. cinerascens* collected during summer and winter, the ^1^H‐NMR profiles were subjected to comparative analysis through the construction of PCA‐X and OPLS‐DA score plots using the SIMCA software v17.0.1. The PCA‐X results (Figure 7a) revealed an *R* ^2^ *X* of 0.947 and *Q* ^2^(cum) of 0.855, indicating the reliability of the model. These results displayed a clustering pattern in which the ‘HC_W_1b’ and ‘HC_W_2b’ winter samples appeared close to the summer ‘HC_S_1b’ sample. However, there was a noticeable seasonal separation between the samples, as indicated by their dispersion away from the central plane of the plot, with greater metabolic differentiation observed within the summer tissues.

Subsequently, the OPLS‐DA (Figure 7b) demonstrated a distinct seasonal separation between the summer and winter samples. The model exhibited *R* ^2^ *X*(cum), *R* ^2^ *Y*(cum) and *Q* ^2^(cum) values = 1, signifying the model's strong predictive capability in distinguishing the seasonal origin of the gonadal tissue samples. The results revealed that the summer samples displayed greater variation within the season than the winter samples, as evidenced by the increased vertical separation observed within the summer samples. This variation could be associated with the spawning cycle of *H. cinerascens*, which engages in sexual reproduction during the summer months, followed by sexual dormancy or regeneration during the colder winter months. Model validation was performed through a permutation plot (Figure S5a), with intercepts at *R* ^2^ = (0.0; 1) and *Q* ^2^ = (0.0; 1). The outcome of this plot was horizontal, and a hierarchical clustering analysis (HCA) (Figure S5b) was carried out as further validation. Furthermore, a cross‐validated ANOVA (CV‐ANOVA) (Table S3) was conducted to confirm the goodness of fit of the OPLS‐DA model, yielding a significant *p* value of 4.23151e − 07, affirming the robustness of the generated model.

The NMR spectral data were visualised through stacked representations (Figure S6) to identify metabolic variations between the gonadal tissues of *H. cinerascens* across summer and winter. Notably, the summer sample ‘HC_S_1b’ displayed significantly higher metabolic concentrations, obscuring the visualisation of the remaining samples. However, the spectral peaks within ‘HC_S_1b’ closely resembled those in the remaining summer gonadal samples and were thus excluded from the overall stack to facilitate visualisation of the other samples.

The spectral comparison revealed distinctive metabolic patterns in the gonadal tissue extracts. For instance, between 0.8 and 1.4 ppm, the summer samples exhibited considerably higher metabolite concentrations and a greater abundance of peaks corresponding to aliphatic or alkyl groups compared to their winter counterparts. A similar trend was observed in the 1.7–2.0 ppm region, where the summer spectra displayed peaks that were either absent or less pronounced in the winter profiles. Moreover, the region between 2.0 and 2.2 ppm featured two multiplets in both seasonal groups, but with higher intensities in the winter samples, potentially corresponding to the amino acids glutamine, glutamate and proline. The 2.8–4.1 ppm region revealed multiple spectral peaks across both seasons, although the summer samples showed taller peak heights. Similarly, both groups displayed a triplet around 3.0 ppm, with higher intensity peaks in the summer samples, whereas a peak around 3.05 ppm was higher in the winter samples. Notably, peaks between 3.1 and 4.0 ppm appeared more pronounced in the winter samples. However, the summer samples exhibited a higher abundance of peaks between 3.4 and 3.5 ppm, seemingly absent in the winter tissues. These can be further seen in the spectral overlap between the ‘S_1b’ and ‘W_1b’ gonadal samples (Figure S6b). Additionally, at around 4.2 ppm, the summer samples displayed a more pronounced multiplet, whereas the winter samples contained a stronger singlet at 4.4 ppm and an additional peak around 4.5 ppm, absent from the summer spectra. The summer samples also contained two doublets between 4.5 and 4.6 ppm, potentially related to sugars, sugar alcohols or vinylic (alkene) protons, whereas the ‘HC_S_2b’ had two peaks around 4.7 ppm, not detected in the other samples. Distinct variations were also noted in the aromatic region. The winter samples displayed a more pronounced singlet around 5.0 ppm, and ‘HC_W_3b’ showed sugar‐related peaks between 5.2 and 5.4 ppm that were largely absent in the other samples. Conversely, the summer spectra featured a pronounced peak at 5.4 ppm, which appeared only in the ‘HC_W_3b’ of the winter samples. Moreover, both seasons exhibited two doublets between 6.0 and 6.2 ppm, though these were more intense in the winter samples. All gonadal spectra displayed a singlet around 6.8 ppm, with an additional small doublet present among the winter profiles. Notably, the winter samples contained more pronounced peaks in the aromatic region between 8.0 and 9.2 ppm, particularly a multiplet and doublet around 8.0 ppm, largely absent in the summer spectra. Additionally, the winter samples displayed minor signals between 8.6 and 8.75 ppm and a low singlet near 9.2 ppm, likely corresponding to trigonelline, which was not observed in the summer samples. Overall, these metabolic variations may be attributed to the reproductive cycles of *H. cinerascens*, underscoring the importance of considering both tissue type and season in metabolic studies of sea cucumbers. These findings provide valuable insights into the metabolic diversity of gonadal tissues and highlight the need for further research on the reproductive biology of *H. cinerascens*.

**FIGURE 8:**
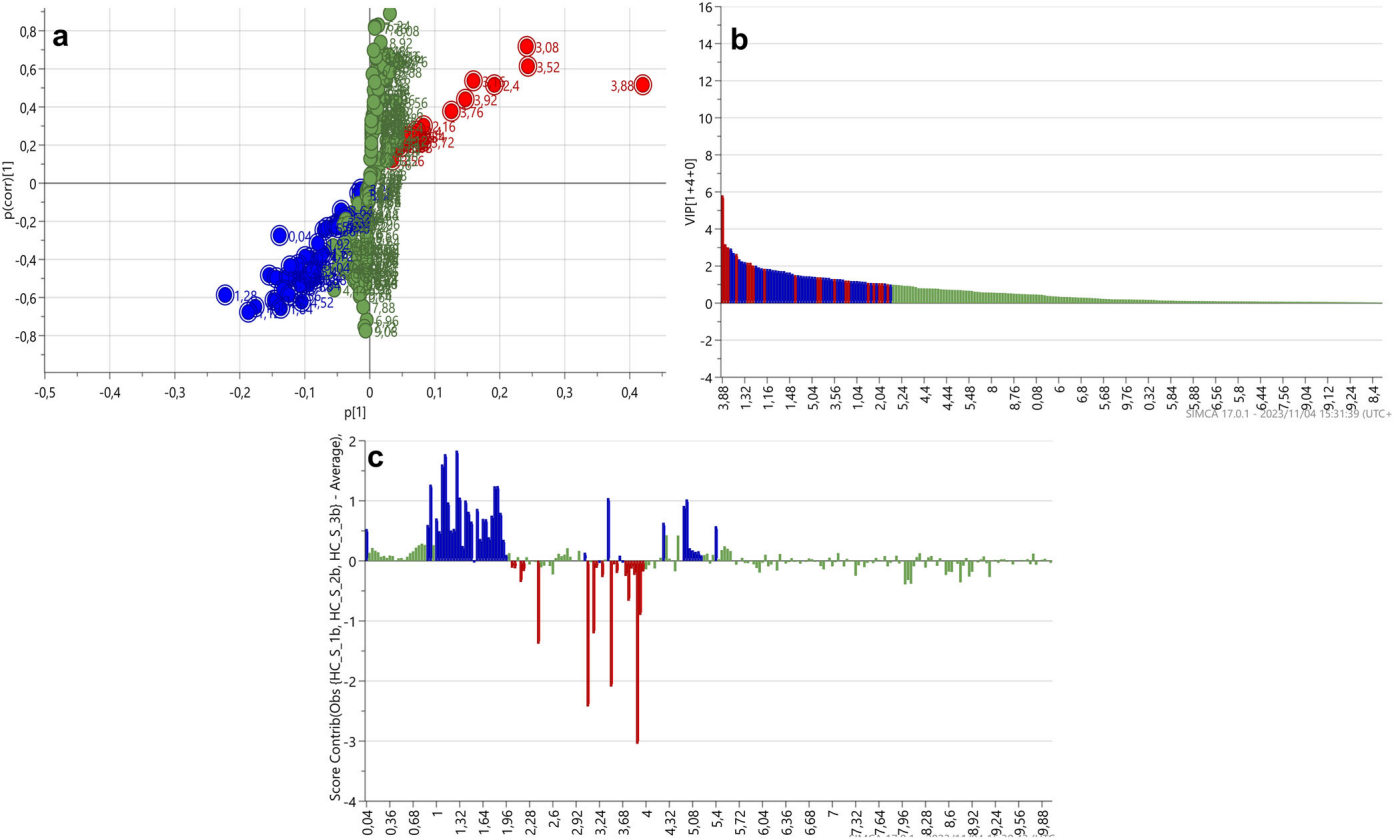
S‐plot (a), VIP score plot (b) and contribution loadings plot (c) showing ^1^H‐NMR spectral regions contributing to the clustering and separation of the gonadal tissues from *Holothuria cinerascens* between summer and winter. Bars above the *X*‐axis (c) showing regions positively associated with the summer gonadal tissue.

An S‐plot and VIP scores plot were generated to determine the spectral regions that influenced the seasonal separation of gonadal tissue samples (Figure 8a,b). Influential chemical shifts were identified from both ends of the S‐plot, and VIP scores ≥1.0. A contribution loadings plot (Figure 8c) revealed that metabolites positively associated with the summer samples included ascorbic acid (*δ* 3.68 and 4.52 ppm), known to play a role in protecting reproductive tissues from harmful free radicals and supporting gametogenesis. Conversely, regions negatively associated with the summer samples (and elevated in winter) included betaine (*δ* 3.4 and 3.84 ppm) and pyruvate (*δ* 2.4 ppm) which are critical for osmoregulation and energy metabolism, respectfully. In addition, other differential metabolites included acetic acid (*δ* ∼1.92 ppm), alanine (*δ* ∼1.48 and 3.76 ppm), glutamate (*δ* ∼2.04, 2.16, 2.4 and 3.76 ppm), arginine (*δ* ∼1.92, 3.2 and 3.80 ppm), lysine (*δ* ∼1.44, 1.88 and 3.72 ppm) and serine (*δ* ∼3.84, 3.92 and 3.96 ppm). Additionally, glucose and galactose (*δ* ∼3.2, 3.4, 3.52, 3.72, 3.76 and 3.84 ppm), valerate (*δ* ∼2.16 ppm), dimethyl sulfone (*δ* ∼3.16 ppm), malonate (*δ* ∼3.16 ppm), methylmalonate (*δ* ∼3.2 ppm), glycerol (*δ* ∼3.52, 3.6 and 3.72 ppm) and myo‐inositol (*δ* ∼3.52, 3.6 ppm) were also elevated in winter gonadal tissues. These seasonal shifts suggest increased osmoregulation and energy mobilisation during winter, with betaine and pyruvate as key discriminating metabolites within the gonadal tissue.

### 4.3 Gut/Mesentery

**FIGURE 9:**
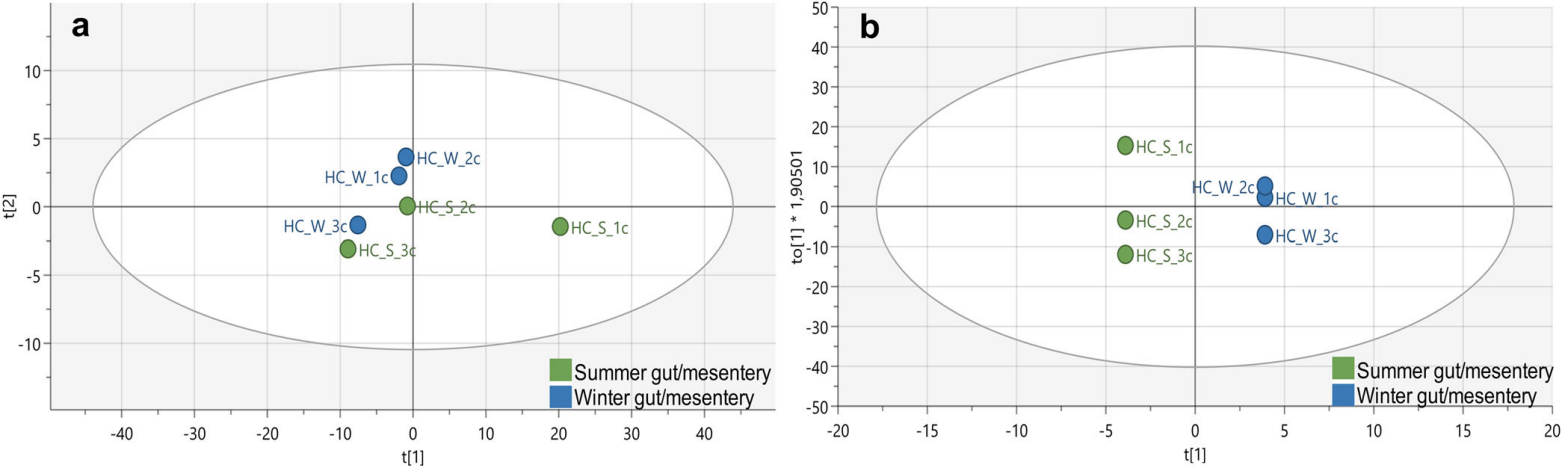
A representation of the PCA‐X (a) and OPLS‐DA score plot (b) from the ^1^H‐NMR spectral data showing the seasonal separation of the gut/mesentery tissue samples from *Holothuria cinerascens*.

The PCA‐X results, depicted in Figure 9a, displayed an *R* ^2^ *X* = 0.971 and *Q* ^2^(cum) = 0.917, revealing a subtle seasonal difference in the gut/mesentery tissue from *H. cinerascens* between summer and winter. The results indicated a greater degree of metabolic variability within the summer samples than in winter. Comparatively, the OPLS‐DA results (Figure 9b) demonstrated a more pronounced seasonal contrast between summer and winter gut/mesentery tissues, mirroring the pattern observed within the gonadal tissue, with the model yielding *R* ^2^ *X*(cum), *R* ^2^ *Y*(cum) and *Q* ^2^(cum) values of 1. A more extensive variability was observed within the summer samples compared to those from winter, which showed closer clustering. This pattern could be linked to seasonal variations in food availability and consumption, with summer possibly providing a greater abundance and diversity of food sources. Consequently, the reduced variation observed in winter samples could be associated with decreased food intake; however, further investigations into the seasonal biology and habitat conditions of this species are warranted. Furthermore, the diversity in gut/mesentery content may be influenced by foreign organisms or particles ingested by the sea cucumber. Model validation was carried out through a permutation plot (Figure S7a), which had intercepts at *R* ^2^ = (0.0; 1) and *Q* ^2^ = (0.0; 1), producing a horizontal result, consistent with the gonadal tissue analysis. As such, a HCA was carried out as further validation (Figure S7b). A CV‐ANOVA (Table S4) was conducted to affirm the model's goodness of fit, resulting in a significant *p* value of 2.79888e − 07, reinforcing the robustness of the model.

NMR stacks, depicted in Figure S8a,b, visually represent the chemical shift regions and highlight the pronounced differences between the gut/mesentery tissues from *H. cinerascens* over summer and winter. Notably, the summer ‘HC_S_3c’ sample exhibited minimal to no chemical shift peaks compared to the other tissue samples, whereas the winter samples exhibited higher spectral peak resolution than their summer counterparts. In the sugar–aliphatic region, the summer ‘HC_S_1c’ sample displayed elevated metabolic concentrations compared to the remaining samples. The winter samples revealed a small triplet at 1.2 ppm, absent in the summer samples, and more pronounced aliphatic proton peak intensities between 1.6 and 2.2 ppm. At 3.0 ppm, the winter samples displayed a distinct triplet, whereas the summer samples displayed either a low triplet or a singlet. Between the 3.1 and 4.0 ppm sugar region, both seasonal groups displayed a variety of spectral peaks likely corresponding to sugars such as xylose and glucose, sugar alcohols and amino acids including proline and serine, with an overall higher resolution in the winter samples. The region between 3.5 and 4.2 ppm appeared more pronounced within the winter samples, with these peaks likely corresponding to glycerol and betaine based on chemical annotation. In the high‐sugar aromatic region, a common spectral pattern was observed between 5.1 and 5.4 ppm likely related to sugar carbohydrates, with minor variations between summer and winter samples. However, the winter samples contained a multiplet and doublet between 5.9 and 6.1 ppm, absent in the summer samples. Significant variations were also evident in aromatic proton signals between 7.9 and 8.8 ppm. All samples, except ‘HC_S_3c’, displayed a multiplet and doublet between 7.9 and 8.05 ppm, with a higher peak intensity observed within the ‘HC_S_1c’. Additionally, the winter samples exhibited a more pronounced singlet and multiplet at 8.25 and 8.55 ppm, respectively, compared to the summer samples.

The observed differences signify distinct metabolic variability between the summer and winter gut/mesentery tissues from *H. cinerascens*, characterised by variations in spectral patterns between the seasons. These distinctions may be related to fluctuations in food sources, their abundance and altered metabolic requirements and processes during different seasons, likely in response to changing environmental conditions and exposure to varying organic matter. Furthermore, variations within the gut microbiota and composition during different seasons may contribute to differing microbial activities, nutritional absorption and the production of exogenous metabolites, all of which may reflect the dynamic metabolic profiles of sea cucumbers over varying seasons.

**FIGURE 10:**
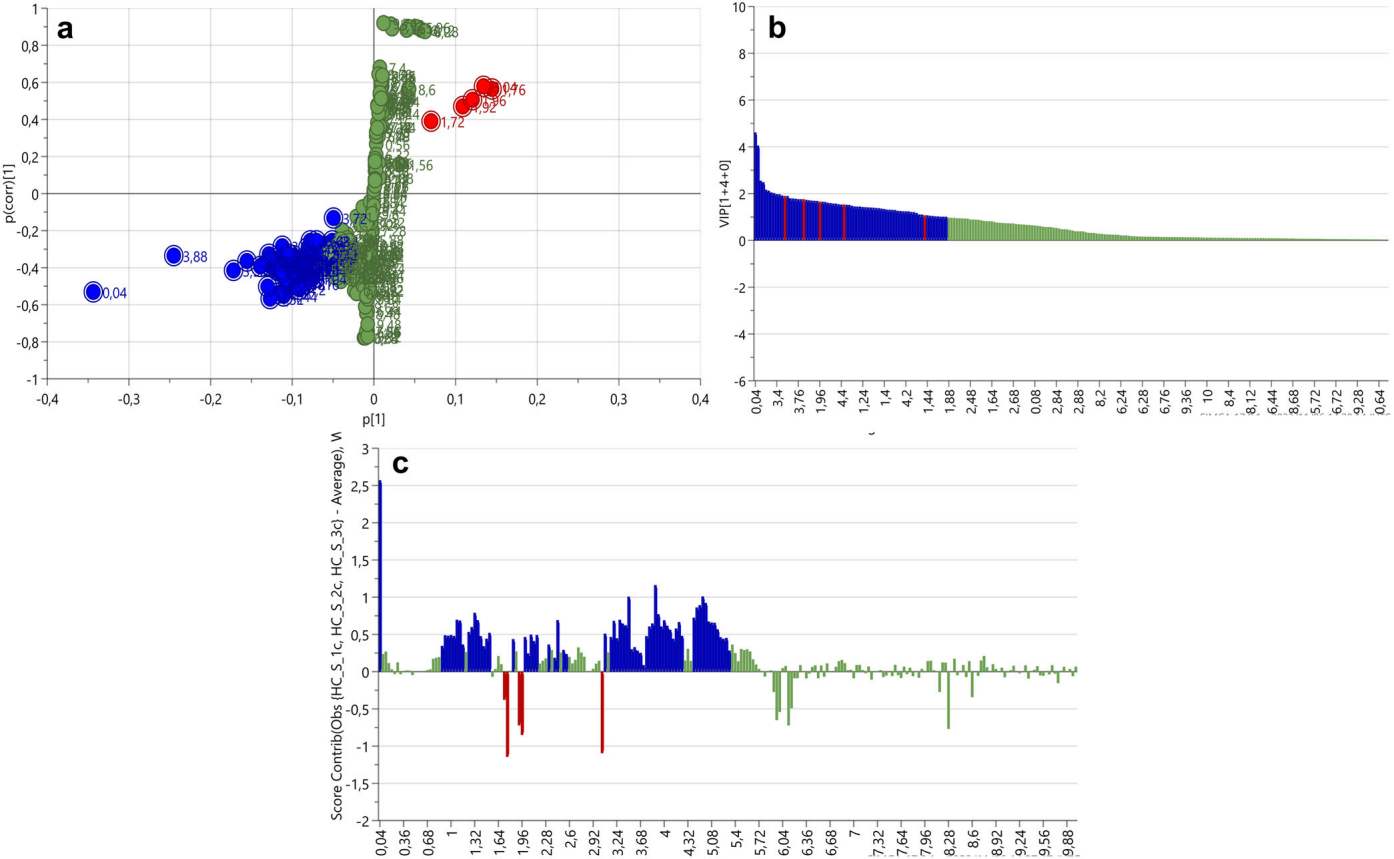
Representation of the S‐plot (a), VIP scores plot (b) and contribution loadings plot (c) from the ^1^H‐NMR spectral data showing influential regions responsible for the clustering and separation of the gut/mesentery tissues from *Holothuria cinerascens* between summer and winter. Bars above the *X*‐axis (c) showing regions positively associated with the summer gut/mesentery tissue.

An S‐plot and VIP scores plot (Figure 10a,b) were generated to identify the spectral regions contributing to the seasonal separation of gut/mesentery tissue samples from *H. cinerascens*, with influential features identified from both ends of the S‐plot and VIP scores ≥1.0. A contribution loadings plot (Figure 10c) further visualised the chemical shift regions associated with the summer and winter samples. Influential regions associated with the summer samples were observed at *δ* 0.04, 1.08, 1.12, 1.28, 1.32, 1.36, 1.52, 2.16, 2.44, 3.16, 3.2, 3.24, 3.4, 3.44, 3.48, 3.52, 3.76, 3.8, 3.84, 3.88, 3.92, 3.96, 4, 4.04, 4.4, 4.44, 4.48, 4.52, 4.56, 5.04, 5.08, 5.12 and 5.16 ppm, with additional regions at *δ* 0.88, 0.92, 0.96, 1, 1.04, 1.16, 1.24, 1.4, 1.44, 1.48, 1.84, 2, 2.04, 2.08, 2.12, 2.32, 2.4, 2.52, 2.56, 3.08, 3.56, 3.6, 3.64, 3.68, 3.72, 4.08, 4.12, 4.16, 4.2, 4.24, 5.2, 5.24, 5.28 and 5.32 ppm contributing to a lesser extent. Negatively associated regions were identified at 1.72, 1.76, 1.92, 1.96 and 3.04 ppm. On the basis of metabolite annotations from Chenomx, literature and online databases, key metabolites contributing to seasonal separation included acetic acid (*δ* ∼1.92 ppm), arginine (*δ* ∼1.92, 3.20 ppm), betaine (*δ* ∼3.24, 3.84 ppm), glycerol (*δ* ∼3.52, 3.60 and 3.72 ppm), lysine (*δ* ∼1.72, 1.92 ppm) and malonate (*δ* ∼3.16 ppm). These compounds were elevated in winter, but also within the ‘HC_S_1c’ sample, likely accounting for the skew towards positive (summer‐associated) loadings and reflecting natural variation and overlapping seasonal fluctuations. Their overall elevation in winter may suggest physiological adjustments in response to cooler conditions. Overall, these findings support tissue‐specific and seasonally influenced metabolic adaptation within the gut/mesentery of *H. cinerascens*.

These findings indicate seasonal variations in metabolite levels within different body tissues of *H. cinerascens*, highlighting potential adaptive responses to changing environmental conditions and tissue‐specific metabolic processes.

Many of the compounds identified have potential functionality in osmoregulation, energy metabolism, chemical defence and reproduction. However, the metabolic pathways of these compounds within *H. cinerascens* remain poorly understood, necessitating further investigation to elucidate their significance. Nonetheless, our study underscores the presence of notable metabolic compounds within *H. cinerascens*, with potential applications in nutrition, natural therapeutics and industrial settings. Future targeted studies are imperative to confirm the presence of these compounds and determine their structural arrangement within larger molecules. Additionally, efforts should focus on isolating compounds using varied analysis techniques and extraction solvents to enhance metabolite extraction concentrations for downstream processing.

### 4.4 UPLC–QTOF–MS Analysis

The metabolites from the summer and winter body tissues of *H. cinerascens* were analysed using UPLC–QTOF–MS (Figure S9), with a summary of the mass spectral results provided in Table 1. Tentative identification of compounds was achieved by comparing mass spectral data with information from online databases and relevant literature. Although several compounds were putatively identified, precise confirmation relies upon the availability of reference standards, targeted fragmentation analyses and accurate, up‐to‐date reference spectra. As such, the characterisation of some compounds remains inconclusive, with several compounds having multiple possible classifications, whereas others remain unknown due to a lack of reference data or standards. Additionally, literature on holothurian metabolites often reflects similar uncertainties, further complicating compound confirmation, especially in untargeted baseline analyses.

**TABLE 1:** Metabolites identified in ESI negative mode by UPLC–QTOF–MS in *Holothuria cinerascens* body tissues (Body wall = ‘BW’, Gonad = ‘G’ and Gut/Mesentery = ‘GM’) during summer and winter.

Rt	Observed *m/z*	Monoisotopic mass	Empirical formula (neutral)	Summer			Winter			Putative compound	References
				BW	G	GM	BW	G	GM		
6.87	209.0834	210.09258	C_8_H_18_O_4_S	X						Dibutyl sulphate Octyl sulphate 6‐Methylheptyl sulphate **Note**: Straight‐chain or branched aliphatic organosulphate	[44, 45] ChemSpider ID: 11738 Or 8547
6.90	195.0620	196.07693	C_7_H_16_O_4_S		X	X	X	X	X	Heptyl hydrogen sulphate	ChemSpider ID: 158845
6.92	209.0836	210.09258	C_8_H_18_O_4_S	X						Aliphatic organosulphate Dibutyl sulphate Octyl sulphate 6‐Methylheptyl sulphate **Note**: Straight‐chain or branched molecules, likely isomers	[44, 45] ChemSpider ID: 11738 Or 8547
7.08	209.0838	210.09258	C_8_H_18_O_4_S	X							
7.16	209.0844	210.09258	C_8_H_18_O_4_S	X							
7.55	209.0842	210.09258	C_8_H_18_O_4_S		X	X					
7.59	209.0840	210.09258	C_8_H_18_O_4_S		X	X					
7.75	209.0839	210.09258	C_8_H_18_O_4_S		X	X					
7.84	209.0838	210.09258	C_8_H_18_O_4_S		X	X					
8.25	237.1141	238.12388	C_10_H_22_O_4_S	X						Diamyl sulphate Decyl sulphate 8‐Methylnoyl hydrogen sulphate **Note**: Straight chain or branched	[45] ChemSpider ID: 13674908 Or 8564
8.30	1199.5144	1200.52337	C_54_H_88_O_27_S	X						Holothurin A1 Holothurin A4 Scabraside D	[16, 43, 46] ChemSpider ID: 29368578
	1199.5177	1199.51915	C_66_H_80_O_19_Na	X						Unknown	
8.39	223.0990	224.10823	C_9_H_20_O_4_S		X	X				2,6‐Dimethylheptyl hydrogen sulphate 6‐Methyloctyl sulphate 7‐Methyloctyl hydrogen sulphate	[45] ChemSpider ID: 23327239 or 141892 PubChem CID: 141892
8.48	1197.5016	1198.50772	C_54_H_86_O_27_S	X						Holothurin A (Arenicolaside A) 17‐Hydroxyfuscocineroside B (Scabraside B) 25‐Hydroxyfuscocineroside B	[16, 43, 46, 47]
	1197.4994	1198.50882	C_70_H_79_O_14_SNa	X						Unknown	
8.72	1181.5083	1182.51280	C_54_H_86_O_26_S	X						Scabraside A 24‐Dehydroechinoside A 17‐Dehydroxyholothurin A (Fuscocineroside C) Fuscocineroside B	[16, 43, 46, 47, 48, 49]
	1181.5016	1182.50693	C_61_H_82_O_21_S	X						Unknown	
8.92	237.1149	238.12388	C_10_H_22_O_4_S					X	X	Diamyl sulphate Decyl sulphate 8‐Methylnoyl hydrogen sulphate **Note**: Straight‐chain or branched molecule	[45] ChemSpider ID: 13674908 Or 8564
8.96	1199.5203	1200.52337	C_54_H_88_O_27_S				X	X	X	Holothurin A1 Holothurin A4 Scabraside D	[16, 43, 46]
9.14	1197.4954	1198.50184	C_61_H_82_O_22_S		X	X	X	X	X	Unknown	
	1197.4998	1197.50100	C_70_H_78_O_14_SNa		X	X	X	X	X	Unknown	
9.30	367.2151	368.22326	C_17_H_36_O_6_S		X	X		X	X	2‐[2‐(Tridecyloxy)ethoxy]ethyl hydrogen sulphate C13‐Alkyl‐2‐ethoxysulphate (C13‐AE2S) **Note**: Straight chain or branched	ChemSpider ID: 2289709 or PubChem CID: 3023544
9.36	1181.5095	1182.51280	C_54_H_86_O_26_S				X			Scabraside A 24‐Dehydroechinoside A 17‐Dehydroxyholothurin A (Fuscocineroside C) Fuscocineroside B	[16, 43, 46, 47, 48, 49]
	1181.5028	1181.50257	C_52_H_86_O_26_SNa				X	X	X	Unknown	
9.39	1181.5133	1182.52806	C_58_H_86_O_23_S		X	X		X	X	Unknown	
	1181.5016	1182.50693	C_61_H_82_O_21_S		X	X		X	X	Unknown	
9.39	1181.5017	1182.51040	C_52_H_87_O_26_SNa		X	X		X	X	Unknown	
9.39	669.2791	670.28704	C_29_H_50_O_15_S	X						32‐([(4‐Methylphenyl)sulfonyl]oxy)‐3,6,9,12,15,18,21,24,27,30‐decaoxadotriacontan‐1‐oic acid	ChemSpider ID: 123961299
9.49	367.2159	368.22326	C_17_H_36_O_6_S					X	X	2‐[2‐(Tridecyloxy)ethoxy]ethyl hydrogen sulphate C13‐Alkyl‐2‐ethoxysulphate (C13‐AE2S) **Note**: Straight chain or branched	ChemSpider ID: 2289709 or PubChem CID: 3023544
9.67	1147.5514	1148.55897	C_59_H_88_O_20_S				X			Unknown	
	1147.5510	1148.55906	C_53_H_89_O_25_Na				X			Unknown **Note**: Co‐elution detected	
9.75	1181.5153	1182.52806	C_58_H_86_O_23_S				X			Unknown	
		1182.51040	C_52_H_87_O_26_SNa				X			Unknown	
	1181.5050	1182.51280	C_54_H_86_O_26_S				X			Scabraside A 24‐Dehydroechinoside A 17‐Dehydroxyholothurin A (Fuscocineroside C) Fuscocineroside B	[16, 43, 46, 47, 48, 49]
9.71	251.1201	252.13953	C_11_H_24_O_4_S		X	X		X	X	Undecyl hydrogen sulphate 4(*R*),8‐Dimethylnonyl sulphate **Note**: Straight‐chain or branched molecule	[45] ChemSpider ID: 109828 ChemSpider ID: 34448657 PubChem CID: 24796507
9.75	251.1299	252.13953	C_11_H_24_O_4_S		X	X		X	X		
9.84	251.1298	252.13953	C_11_H_24_O_4_S		X	X		X	X		
10.11	669.2792	670.28704	C_29_H_50_O_15_S				X			32‐([(4‐Methylphenyl)sulfonyl]oxy)‐3,6,9,12,15,18,21,24,27,30‐decaoxadotriacontan‐1‐oic acid	ChemSpider ID: 123961299
10.43	671.2966	672.30269	C_29_H_52_O_15_S				X			Unknown	
10.60	1177.5172	1178.52376	C_48_H_90_O_30_S		X	X				Unknown	
	1177.5054	1178.51202	C_62_H_82_O_20_S		X	X				Unknown	
	265.1478	266.15518	C_12_H_26_O_4_S		X	X				Lauryl sulphate/Dodecyl hydrogen sulphate **Note**: Straight‐chain or branched molecule	[50] ChemSpider ID: 8448 PubMed CID: 8778
10.60	859.3828	860.38642	C_41_H_64_O_17_S				X	X	X	Holothurin B (Axilogoside A) Holothurin B4 Nobiliside B Nobiliside II (Ananaside C)	[16, 43, 46, 47, 49, 51, 52, 53] ChemSpider ID: 25052543 PubChem CID: 23674754
	859.3779	859.37619	C_39_H_64_O_17_SNa				X	X	X	Unknown	
10.71	711.2894	712.29775	C_52_H_40_O_3_				X			Unknown	
		712.29534	C_50_H_41_O_3_Na				X			Unknown	
10.88	713.3018	714.31326	C_31_H_54_O_16_S				X			35‐([(4‐Methylphenyl)sulfonyl]oxy)‐3,6,9,12,15,18,21,24,27,30,33‐undecaoxapentatriacontan‐1‐oic acid	[43] ChemSpider ID: 123961295
10.96	843.3841	844.39151	C_41_H_64_O_16_S				X			Holothurin B3 24‐Dehydroechinoside B	[16, 43, 47, 51]
11.07	655.2979	656.30778	C_29_H_52_O_14_S	X			X			Unknown	
11.25	613.2875	614.29721	C_27_H_50_O_13_S				X			Unknown	
11.65	655.2977	656.30778	C_29_H_52_O_14_S				X			Unknown	
11.76	655.3008	656.30778	C_29_H_52_O_14_S				X			Unknown	
11.78	619.2897	620.29613	C_34_H_45_O_9_Na					X	X	Unknown	
12.05	619.2881	620.29613	C_34_H_45_O_9_Na					X	X	Unknown	

*Note*: ‘X’ indicates compound presence. Average percentage error = 0.0067%.

Abbreviation: UPLC–QTOF–MS, ultra performance liquid chromatography quadruple time‐of‐flight mass spectrometry.

The analysis revealed a rich diversity of metabolites across tissues, demonstrating distinct seasonal variations in metabolite profiles between summer and winter body tissues (Table 1, Figure S9). Notably, a clear separation between the body wall and the gonad and gut/mesentery tissues was observed, corroborating the OPLS‐DA results illustrated in Figures 1 and 2. Although seasonal differences were evident, the overall distribution of metabolites between summer and winter was relatively balanced. Metabolites also exhibited significant tissue specificity, with several compounds found exclusively in the body wall or shared between the gonad and gut/mesentery tissues. The body wall tissues displayed a higher metabolite diversity, aligning with the ^1^H‐NMR results summarised in Table S2.

Among the identified compounds were potential saturated aliphatic organosulphates and triterpene glycosides, with subtle mass spectral differences suggesting a high degree of isomerism within this species. This compound isomerism presents additional challenges for confirmation, as it requires advanced analytical techniques to distinguish between structural variants. Many of the identified saponins were localised to the body wall, potentially reflecting elevated stress levels, as saponins are known to play a critical role in the chemical defence mechanisms of sea cucumbers [11, 19, 42, 43]. Although several compounds in Table 1 have been previously reported in echinoderms, their exact identities remain uncertain due to conflicting literature, the potential for multiple matches and insufficient spectral data for precise validation.

Interestingly, several unknown compounds were detected, particularly within the gonad and gut/mesentery tissues, suggesting potential roles in reproductive and metabolic processes. Whether these compounds originate from endogenous metabolic processes, gut microbiota or dietary sources, such as detritus, microalgae or phytoplankton, remains unclear. It is essential to note that the spectral data were evaluated based on broad parameters set by the analytical software, and definitive identification will require further studies employing targeted fragmentation analysis and reference standards. Despite these challenges, the results underscore the significant bioactive potential of *H. cinerascens*. These findings highlight the necessity of continued research to elucidate the origins, chemical structures and biological properties of these metabolites. Additionally, potentially valuable biomolecules in this species support the expansion of aquaculture practices to include alternative species. Such an approach could promote sustainable utilisation of biological resources while mitigating the ecological and economic pressures associated with overharvesting high‐value species.

## 5 Discussion

The metabolite, nutritional and chemical composition of an organism can vary depending on numerous endogenous and exogenous factors [3, 7, 8, 11, 37, 40, 54]. In sea cucumbers, different tissues serve diverse physiological roles, contributing to variations in their metabolic profiles. Understanding these tissue‐specific and seasonal metabolic shifts is essential to assess how pollution, climate change, stress, environmental variation and diet influence their metabolic composition and nutritional quality. This knowledge is particularly relevant for conservation efforts and optimising aquaculture practices aimed at harnessing sea cucumber resources for nutritional, commercial and pharmaceutical purposes. This study presents the first untargeted metabolomic profiling of *H. cinerascens* from the eastern coast of Southern Africa. The aim was to establish a baseline metabolic profile and investigate variation across three body tissues (body wall, gonad and gut/mesentery) between summer and winter using ^1^H‐NMR and UPLC–QTOF–MS. Our findings revealed distinctive tissue‐ and season‐specific metabolic variation, offering new insights into the metabolic activities and potential functional roles of metabolites within *H. cinerascens*.

The body wall demonstrated the highest abundance of several amino acids—including taurine, threonine, alanine and glycine—alongside short‐chain fatty acids, dicarboxylic acids and dimethyl sulfone (an organosulphur compound). The higher abundance of monosaccharides and osmolytes during summer (Figure S2, Table S2) suggests roles in osmoregulation and defence in response to environmental fluctuations. UPLC–QTOF–MS analysis further revealed the tentative presence of multiple triterpene glycosides. These included holothurin A, A1, A4, B, B3 and B4; scabraside A and D; 17‐hydroxyfuscocineroside B (scabraside B); 25‐hydroxyfuscocineroside B; 24‐dehydroechinoside A; fuscocineroside B; 17‐dehydroxyholothurin A (fuscocineroside C); nobiliside B and nobiliside II (ananaside C) (Table 1). These compounds, also called saponins, are known to support chemical defence mechanisms against predation and environmental stress responses, with many of these compounds demonstrating pharmacological properties, including anticancer, anti‐inflammatory and antimicrobial effects, with species in more exposed habitats, such as *H. cinerascens*, often exhibiting higher saponin levels [11, 19, 20, 42]. Sea cucumbers lacking Cuvierian tubules, including several *Holothuria* species, may rely on higher levels of oxidised sulphated and non‐sulphated saponins for defence and stress resistance, alongside concealment behaviours evolved by these species for survival [19, 20]. In addition, similar to *Apostichopus japonicus, H. cinerascens* shows high sensitivity in its internal tissues and employs evisceration as a defence strategy against environmental stress and excessive handling [19, 55]. Our results also revealed a greater abundance of amino acids in the body wall, supporting findings that this tissue is likely a rich source of peptides that may present varying bioactivities, including antimicrobial and antihypertensive properties [7]. Compounds, such as isovalerate, isobutyrate and dimethyl sulfone, were minimally represented within the gonad and gut/mesentery, indicating their localised importance within the body wall.

In contrast, the gut/mesentery tissues showed higher levels of glycerol, ascorbic acid, galactose, xylose, lysine, proline and serine—metabolites associated with digestion, energy metabolism and regulation of cellular osmolarity. Notably, compounds like glycerol, glutamine, proline, taurine, glycine and glucose are often called ‘compatible osmolytes’ because they can be produced in high concentrations within cells without disturbing cellular function [56, 57, 58]. These metabolites may stabilise proteins and regulate cellular osmolarity to prevent water loss in organisms exposed to high salinity environments [38, 56, 57, 58]. However, some metabolites may originate from symbiotic microorganisms or dietary sources, such as phytoplankton [56, 57, 59]. This raises uncertainty about whether several of these compounds are endogenously produced or acquired via diet or symbionts.

Although the gonadal tissue exhibited the lowest overall metabolite abundance, it contained elevated levels of betaine, an important osmoregulatory compound that can assist marine organisms in maintaining cellular structure in their high salinity environments and pyruvate, a key intermediate in energy metabolism [38, 60]. Pyruvate's role in energy metabolism is critical in supporting the energy demands of reproductive activities, and seasonal increases in pyruvate levels during winter may reflect its role in mobilising stored energy when food availability is limited. The roles of betaine and pyruvate in osmoregulation and energy metabolism suggest their importance in reproductive processes, highlighting the need for further investigations into their specific metabolic properties and functional roles within *H. cinerascens*. Notably, compounds, such as glutamate, acetic acid and myo‐inositol, appeared evenly distributed across the body tissues, suggesting potential shared metabolic functions or regulatory roles. These findings reveal specific metabolic adaptations of *H. cinerascens* that may inform future applications in sustainable aquaculture and natural product discovery. The identification of several triterpene glycosides, particularly within the body wall of *H. cinerascens*, along with numerous unidentifiable metabolites, underscores the potential pharmacological significance of its metabolites. This highlights the need for further research into this species, previously classified as ‘low‐value’ [14], to fully quantify and characterise these metabolites to explore its biomedical and biotechnological potential.

Seasonal comparisons within *H. cinerascens* tissues revealed distinct metabolic shifts. In the body wall, metabolites associated with energy metabolism, osmoregulation and defence were more abundant during summer (Table S2). In contrast, myo‐inositol, malonate, methylmalonate, taurine, dimethyl sulfone and fatty acids (valerate and butyrate) were elevated in winter. Compounds, such as malonate, methylmalonate and dimethyl sulfone (MSM), are rarely reported in sea cucumbers. Malonate is known to contribute to enzymatic reactions and the conversion of oxalacetate to pyruvate in the TCA cycle, while also acting as a cytotoxic inhibitor of the oxidation of succinate to fumarate in the Krebs cycle [61]. Dimethyl sulfone is a highly stable organosulphur compound that is widely used as a supplement due to its anti‐inflammatory and liver‐protecting properties and may play a role in contributing to the elasticity and regeneration properties of sea cucumbers [62]. Similar seasonal shifts observed in the gonadal and gut/mesentery tissues suggest that *H. cinerascens* modulates metabolic activity in response to environmental cues and possibly reproductive timing, where higher metabolite concentrations may support regeneration and energy demands (Table S2). However, further research is warranted to quantify these metabolites, elucidate their origins, functional roles and associated metabolic pathways.

The detection of various aliphatic organosulphates in this study—though rarely reported in sea cucumbers—has been documented in other echinoderms, sponges and ascidians [45]. Although their ecological roles remain largely unknown, these compounds have demonstrated several promising antithrombotic, antiproliferative, antibacterial and antifungal properties [45]. Furthermore, the increased abundance of various amino acids in the body wall during winter supports the potential for peptide synthesis, aligning with studies that highlight the high protein content of sea cucumbers and bioactive peptide potential of the holothurian body wall [7, 12, 25, 63, 64].

However, the metabolic composition of sea cucumber tissues, including *H. cinerascens*, can be influenced by dietary composition, gut microbiota and environmental factors [22]. Environmental pollution could significantly impact water quality and food availability, further impacting metabolite production in marine organisms. Sea cucumbers in suboptimal habitats may exhibit reduced nutritional potential or increased stress‐related metabolites due to resource allocation towards immune responses or protective mechanisms, such as dormancy during stress periods. Pollution, habitat destruction and pathogenic microorganisms represent significant stressors, particularly for marine species inhabiting coastal or estuarine environments. Species in these regions are at greater risk of exposure to sewage outlets, industrial waste disposal, chemical spills and harmful pathogens than those in deeper sea habitats [11]. As such, this study raises concerns about environmental pollution at Park Rynie, which has an active sewerage outlet and experienced a chemical spill prior to this study, which may have impacted the metabolic profile of *H. cinerascens*. The detection of compounds like dodecyl hydrogen sulphate (lauryl sulphate) raises concerns about marine contamination from surfactants commonly used in wastewater treatments, industrial processes and household applications [50]. This finding underscores the urgent need for comprehensive environmental assessments to evaluate their impact on marine ecosystems and holothurian metabolites.

Despite these challenges, many compounds identified in *H. cinerascens* likely play roles in osmoregulation, energy metabolism, chemical defence and reproduction, reflecting the species’ complex biochemical profile. Unidentified metabolites in this study may represent novel metabolites, underscoring the need for advanced analytical techniques and targeted compound isolation and fragmentation analyses to elucidate their metabolic pathways and functional roles. Additionally, the chemical complexity and variability of metabolites suggest that some metabolites may have remained undetected due to methodological limitations, emphasising the importance of further research utilising alternative analytical techniques and extraction methods to comprehensively understand the metabolome of *H. cinerascens*. Further research should prioritise increased sample sizes to enable improved visualisation of class separation, along with targeted analyses, incorporating fragmentation and quantification analyses to enhance our understanding of the metabolic composition within *H. cinerascens* by allowing software tools to match the mass spectral data with proposed chemical structures. Investigating the metabolic pathways associated with these compounds will offer valuable insights into the physiological adaptations of *H. cinerascens*, contributing to the expansion of sea cucumber research. Such efforts will also support the sustainable incorporation of alternative sea cucumber species into aquaculture practices.

## 6 Conclusion

This study presents the first untargeted metabolomic analysis of *H. cinerascens* from KwaZulu‐Natal, South Africa, using ^1^H‐NMR and complementary full‐scan UPLC–QTOF–MS to evaluate the influence of tissue type and seasonal variation on metabolite diversity. The findings provide novel insights into the metabolic composition and physiological adaptations of *H. cinerascens*. Among the three tissues analysed—body wall, gonad and gut/mesentery—the body wall exhibited the highest metabolite diversity. ^1^H‐NMR detected several amino acids and key sugars, such as xylose and glucose, whereas UPLC–QTOF–MS tentatively identified triterpene glycosides—including holothurin A, scabrasides and nobilisides—predominantly in summer samples, suggesting roles in heat stress mitigation and defence mechanisms. Seasonal variation was most pronounced in the gonad and gut/mesentery, likely reflecting reproductive activity and changes in energy metabolism. Notably, osmoregulatory compounds, such as glycerol and betaine, were prominent in all tissues but especially the gut/mesentery and gonadal tissues, indicating potential physiological adaptations to osmotic stress in their harsh marine environments. Compound identification was limited by structural isomerism and gaps in metabolite databases, leaving several compounds unassigned. This underscores the need for targeted quantification, compound isolation and fragmentation studies to confirm chemical structures and determine origin. Moreover, while effective for polar compounds, the aqueous methanol extraction method may have excluded more non‐polar metabolites, such as lipids, pointing to a key limitation of solvent selection in metabolomic workflows. Therefore, future studies should explore complementary extraction protocols utilising alternative solvents to comprehensively characterise the metabolome of *H. cinerascens*. Despite sample size limitations and the lack of spectral data on holothurian metabolites, this study provides foundational insights into the metabolite composition of *H. cinerascens* and the effects of tissue type and season on its metabolic profile. Future research should prioritise compound isolation and characterisation, integrate transcriptomic and genomic data to elucidate metabolic pathways and the functional roles of key metabolites, and clarify the origin of metabolites, whether they are endogenously synthesised or exogenously acquired. Studies incorporating controlled environmental conditions will also enhance our understanding of sea cucumber physiology and support applications in sustainable aquaculture, environmental monitoring and marine natural product discovery, particularly within underexplored Southern African species.

## Conflicts of Interest

The authors declare no conflicts of interest.

## Supporting information




**Supporting File 1**: cbdv70717‐sup‐0002‐SuppMat.pdf

## Data Availability

The data that support the findings of this study are available in the Supporting Information of this article.
