# Supplementary material for: Untargeted Metabolomics Reveals Seasonal and Tissue‐Specific Metabolic Shifts in Holothuria cinerascens
Source: Chem Biodivers. 2025 Dec 5;23(2):e01499. doi: 10.1002/cbdv.202501499 (PMC12860522; doi:10.1002/cbdv.202501499)
Supplement: Supplementary file 1 — Supporting File 1: cbdv70717‐sup‐0002‐SuppMat.pdf [file CBDV-23-e01499-s001.pdf]

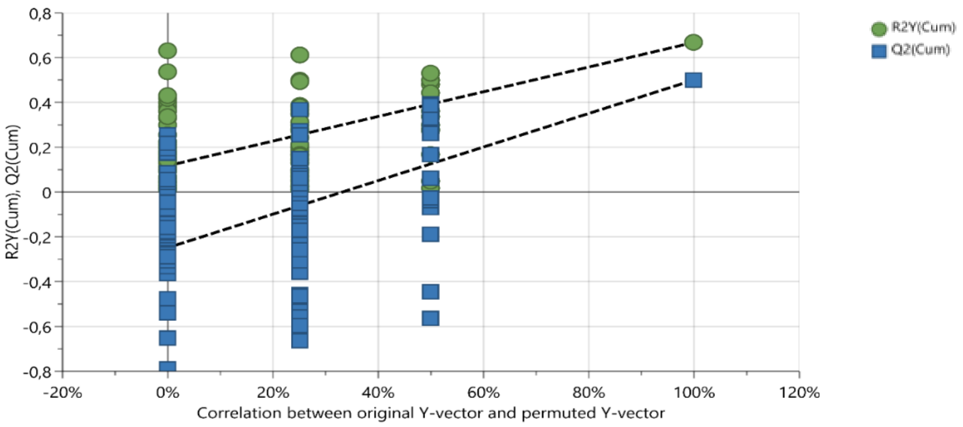

Supplementary Figure 1. Permutation validation tests for the overall body tissue comparisons between summer and winter tissues from *H. cinerascens*. Intercepts for the permutation validation were R^2 = (0.0; 0.117) and Q^2 = (0.0; -0.249)

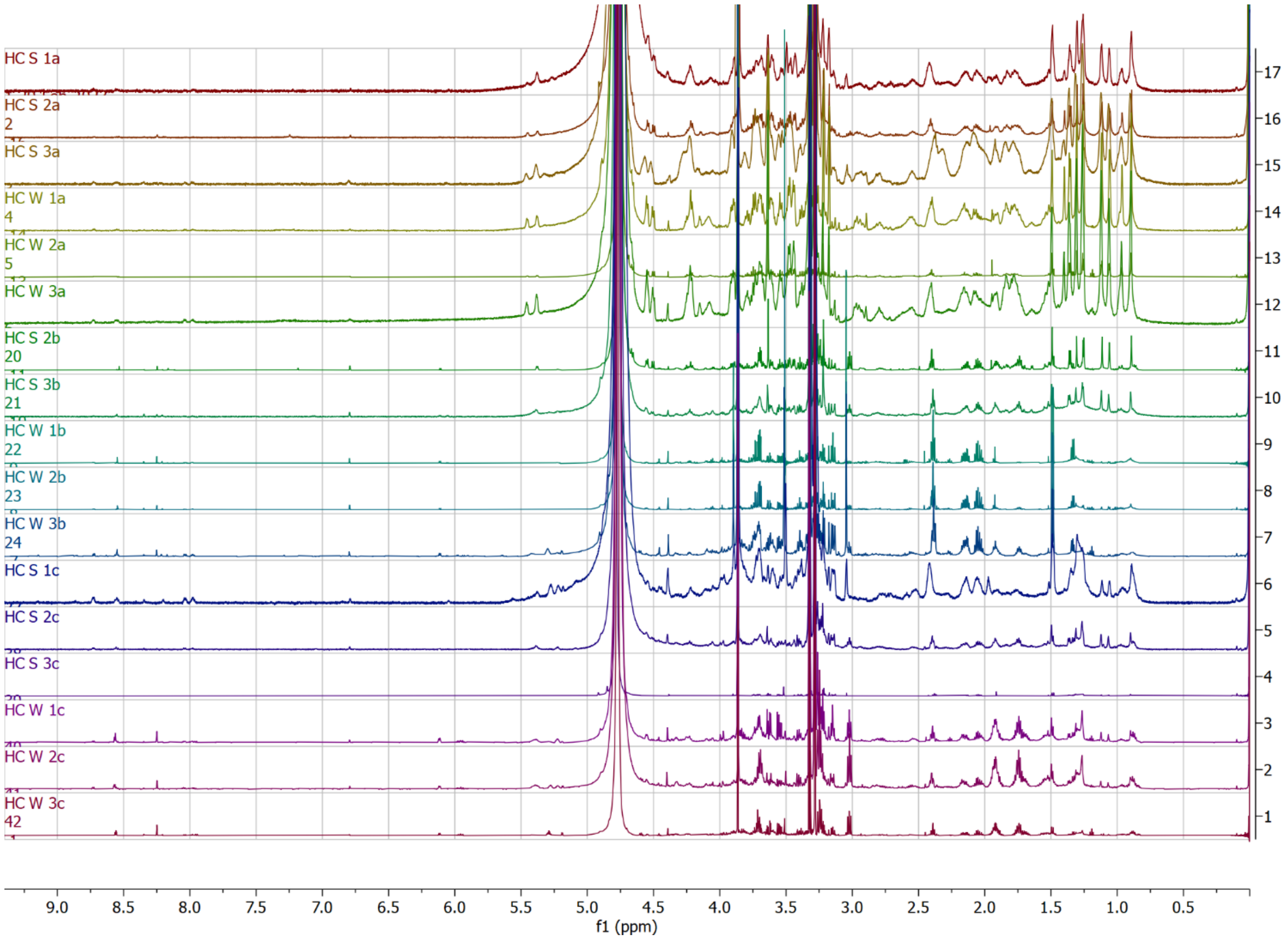

Supplementary Figure 2. NMR spectral stack showing the differentiated chemical shift regions between the various body tissues from *H. cinerascens*. With the letters "a" representing body wall, "b" = gonad, "c" = gut/mesentery, "S" = summer and "W" = winter.

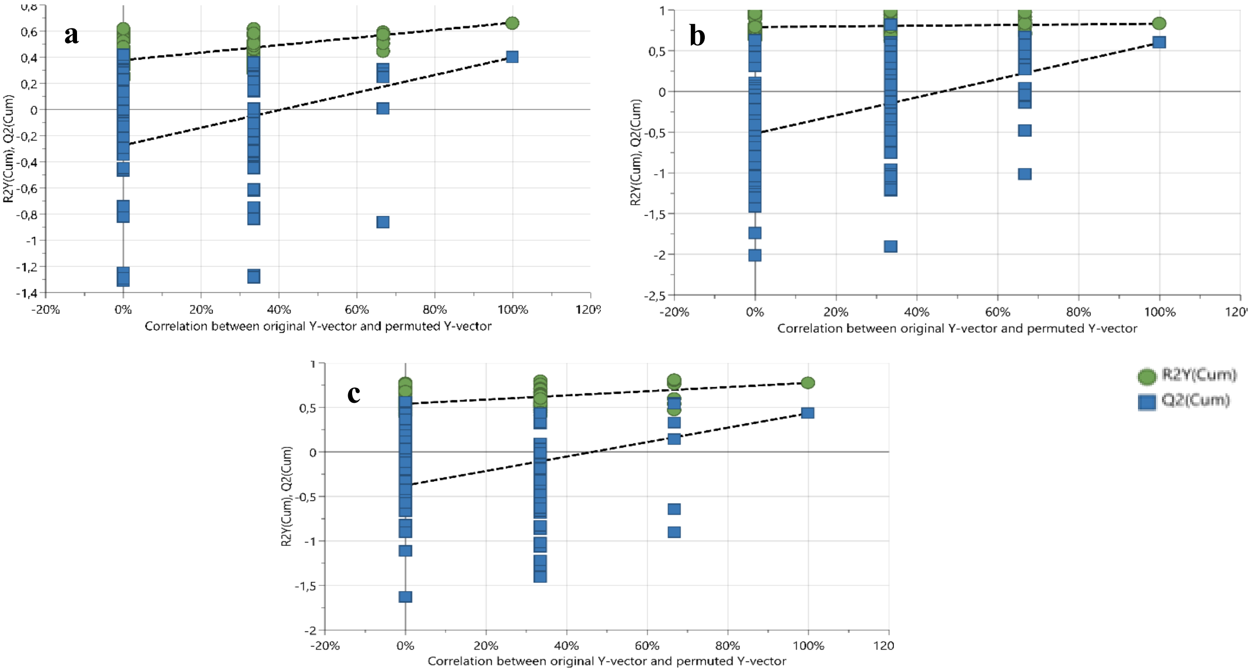

Supplementary Figure 3. Permutation validation results for the body wall vs gonad (a), body wall vs gut/mesentery (b), and gonad vs gut/mesentery (c) tissue comparisons from *H. cinerascens*. Intercepts for the permutation validation tests were: (a) R^2 = (0.0; 0.397) and Q^2 = (0.0; -0.273), (b) R^2 = (0.0; 0.79) and Q^2 = (0.0; -0.517), and (c) R^2 = (0.0; 0.541) and Q^2 = (0.0; -0.377).

Supplementary Table 1. ^{1}H-NMR identified metabolites between the body wall, gonad, and gut/mesentery tissue from *Holothuria cinerascens.*

Metabolite	^{1}H-NMR Chemical shifts (ppm)	Chenomx (ppm)	Human Metabolome Database	Reference Chemical Shift (ppm)	Literature
Acetic acid/Acetate	1.91	1.9	1.91	1.91	[1]
Alanine	1.47<br>3.76	1.5<br>3.8	1.47<br>3.77	1.47<br>3.77	[2-4]
Arginine	1.66<br>1.72<br>1.88<br>1.92<br>3.22<br>3.78	1.6<br>1.7<br>1.9<br>1.9<br>3.2<br>3.8<br>6.7<br>7.2	1.53<br>1.59<br>1.86<br>3.17<br>3.77	1.68<br>1.90<br>3.23<br>3.74	[2, 4]
Ascorbic acid	3.70<br>3.74<br>4.05<br>4.52	3.7<br>3.8<br>4.0<br>4.5	3.44<br>3.73<br>4.72	4.03	[5]
Betaine	3.25<br>3.84	3.3<br>3.9	3.25<br>3.89	3.25<br>3.89	[4, 6]
Butyrate/Butyric acid	0.87<br>1.51<br>2.14	0.9<br>1.5<br>2.2	0.88<br>1.54<br>2.14	0.86<br>2.18	[7]
D-Glucose	3.23<br>3.38<br>3.40<br>3.45<br>3.48<br>3.52<br>3.70<br>3.72<br>3.75<br>3.82<br>3.83<br>3.88<br>4.64<br>5.21	3.2<br>3.4<br>3.4<br>3.5<br>3.5<br>3.5<br>3.7<br>3.7<br>3.8<br>3.8<br>3.8<br>3.9<br>4.6<br>5.2	3.39<br>3.52<br>3.72<br>3.81<br>4.03<br>5.21	3.23<br>3.40<br>3.46<br>3.52<br>3.73<br>3.82<br>3.88<br>4.63<br>5.22	[4]
Dimethyl sulfone	3.16	3.1	3.14	3.14	[1]
D-Xylose	3.21<br>3.31<br>3.42<br>3.48<br>3.60<br>3.61<br>3.66<br>3.69<br>3.88<br>4.52<br>5.19	3.2<br>3.3<br>3.4<br>3.5<br>3.6<br>3.6<br>3.7<br>3.7<br>3.7<br>3.9<br>4.6<br>5.2	3.52<br>3.68<br>3.68<br>3.87<br>4.00<br>4.63	3.68-3.72<br>4.07-4.10<br>4.08-4.11<br>4.29-4.33<br>4.43-4.46<br>4.78	[8-9]
Galactose	3.47<br>3.64<br>3.69<br>3.71<br>3.73<br>3.73<br>3.76<br>3.79<br>3.84<br>3.93<br>3.98<br>4.07<br>4.57<br>5.27	3.5<br>3.6<br>3.7<br>3.7<br>3.7<br>3.7<br>3.8<br>3.8<br>3.8<br>3.9<br>4.0<br>4.1<br>4.6<br>5.3	3.52<br>3.74<br>3.82<br>3.95<br>4.12<br>5.21	3.81<br>4.07	[3]

Glutamate	2.03<br>2.13<br>2.37<br>2.41<br>3.76	2.0<br>2.1<br>2.3<br>2.4<br>3.8	2.06<br>2.11<br>2.32<br>2.36<br>3.75	2.08<br>2.34<br>3.74	[4]
Glutamine	2.11<br>2.14<br>2.38<br>2.40<br>3.76	2.1<br>2.1<br>2.4<br>2.5<br>3.8<br>6.9<br>7.6	2.12<br>2.42<br>2.46<br>3.76	2.12<br>2.44<br>3.70	[7]
Glycerol	3.53<br>3.61<br>3.71	3.6<br>3.6<br>3.8	3.43<br>3.60<br>3.61<br>3.85	3.54<br>3.62	[7]
Glycine	3.55	3.6	3.55	3.57	[3-4, 8]
Isobutyrate/Isobutyric acid	1.04<br>2.38	1.1<br>2.4	1.21<br>2.59	1.07	[10]
Isovalerate/Isovaleric acid	0.89<br>1.94<br>2.03	0.9<br>1.9<br>2.1	0.91<br>0.94<br>1.94<br>2.04	0.88	[5]
Lysine	1.43<br>1.50<br>1.71<br>1.87<br>1.91<br>3.00<br>3.75	1.4<br>1.5<br>1.7<br>1.9<br>1.9<br>3.0<br>3.8	1.43<br>1.72<br>1.90<br>2.12<br>3.01<br>3.74	1.46<br>1.70<br>1.89<br>3.01<br>3.74	[2]
Malonate	3.13/3.14	3.1	3.11	3.11	[3]
Methylmalonate	1.24<br>3.18	1.2<br>3.2	1.23<br>3.16	1.21	[5, 10]
Myo-Inositol	3.27<br>3.52<br>3.60<br>4.04	3.3<br>3.5<br>3.6<br>4.1	3.27<br>3.54<br>3.54<br>3.61<br>3.61<br>4.05	3.52<br>3.60	[5]
Proline	1.99<br>2.03<br>2.05<br>2.34<br>3.32<br>4.12	2.0<br>2.0<br>2.1<br>2.3<br>3.3<br>3.4<br>4.1	1.47<br>1.75<br>2.20<br>2.99<br>3.40<br>3.57	1.99<br>2.06<br>2.33<br>3.33<br>3.40<br>4.12	[11, 2]
Pyruvate	2.36	2.4	2.36	2.40	[1]
Serine	3.84<br>3.94<br>3.97	3.8<br>3.9<br>4.0	3.84<br>3.94<br>4.00	3.84<br>3.96	[4]
Taurine	3.25<br>3.42	3.5<br>3.4	3.25<br>3.41	3.27<br>3.43	[3]
Threonine	1.31<br>3.60<br>4.21	1.3<br>3.6<br>4.3	1.32<br>3.58<br>4.24	1.33<br>3.58<br>4.25	[4]
Trigonelline	4.44<br>8.09<br>8.84<br>8.84<br>9.13	4.4<br>8.1<br>8.8<br>8.8<br>9.1	4.43<br>8.07<br>8.83<br>9.11	8.08<br>8.84<br>9.13	[12-14]

Valerate/Valeric acid	0.86<br>1.31<br>1.51<br>2.15	0.6<br>1.3<br>1.5<br>2.2	0.88<br>1.33<br>1.51<br>2.17	1.20<br>1.56	[10]

Supplementary Table 2. Average seasonal variability of metabolites identified from ^{1}H-NMR between and within the body tissues of *Holothuria cinerascens.*

**Metabolite**	**Higher in:**	**Season higher**		
		**Body wall**	**Gonad**	**Gut/Mesentery**
Acetic acid/Acetate	BW=Gonad=Gut	E	W	W
Alanine	BW>Gut>Gonad	S	W	E
Arginine	BW=Gut>Gonad	W	W	W
Ascorbic acid	Gut>BW=Gonad	S-LOW	S-LOW	E
Betaine	Gonad>Gut>BW	E	W	W
Butyrate/Butyric acid	BW>Gut>Gonad	W	E-LOW	W
D-Glucose	BW=Gut>Gonad	S	W	E
Dimethyl sulfone	BW>Gonad=Gut	W	W-LOW	E-LOW
D-Xylose	Gut>BW>Gonad	S	E	E
Galactose	Gut>BW>Gonad	S	W	W
Glutamate	BW=Gonad>Gut	S	W	W
Glutamine	BW=Gonad=Gut	E-LOW	W-LOW	E-LOW
Glycerol	Gut>BW=Gonad	S	W	W
Glycine	BW>Gut>Gonad	S	E-LOW	W
Isobutyrate/Isobutyric acid	BW>Gonad=Gut	S	E-LOW	S-LOW
Isovalerate/Isovaleric acid	BW>Gonad=Gut	E	E-LOW	S-LOW
Lysine	Gut>Gonad>BW	S-LOW	W	W
Malonate	BW>Gonad>Gut	W	W	W
Methylmalonate	BW>Gut>Gonad	W	S	S
Myo-Inositol	BW=Gonad=Gut	W	W	W
Proline	Gut>BW=Gonad	E-LOW	E	E
Pyruvate	Gonad>BW>Gut	S	W	E
Serine	Gut>Gonad>BW	E-LOW	W	S
Taurine	BW>Gut>Gonad	W	E	S
Threonine	BW>Gonad=Gut	E	E	W
Trigonelline	Gonad>Gut=BW	E-LOW	W-LOW	E-LOW
Valerate/Valeric acid	BW>Gut>Gonad	W	W	W

"BW" = body wall; "W" = winter; "S" = summer; "E" = even.

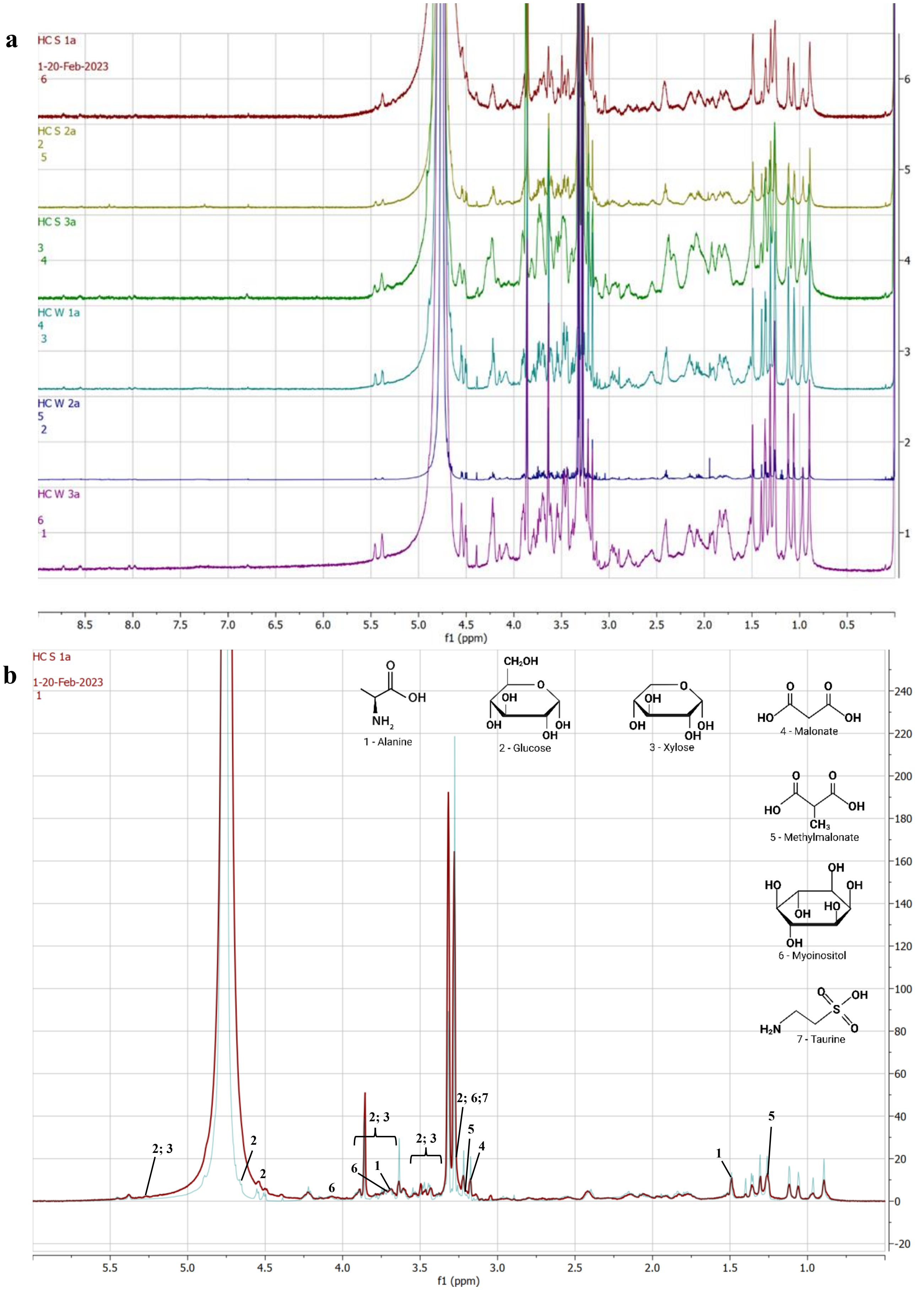

Supplementary Figure 4. (a) NMR spectral stack highlighting the chemical shift regions within the body wall tissue comparison from *H. cinerascens* between summer ("S") and winter ("W"). **(b)** Labelled NMR spectral overlay of *"HC_S_1a"* and *"HC_W_1a"* samples reflecting key differentiating metabolites among summer (red) and winter (blue) body wall tissue extracts. 1 – alanine, 2 – glucose, 3 – xylose, 4 – malonate, 5 – methylmalonate, 6 – myo-inositol, 7 – taurine. Structures created in BioRender. Okpeku, M. (2025) https://BioRender.com/0012vtl

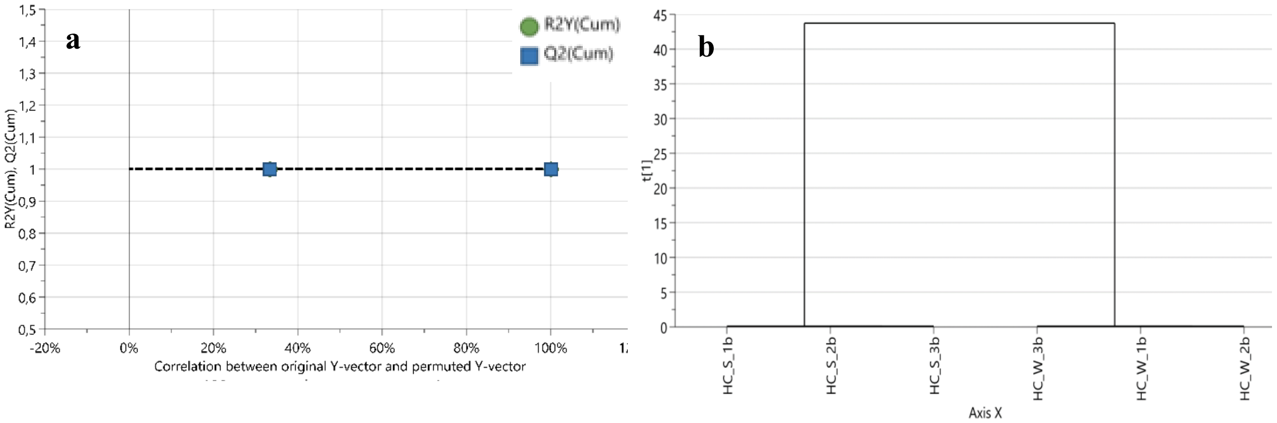

Supplementary Figure 5. Permutation validation (a) and Hierarchical Cluster Analysis (HCA) (b) for the gonadal tissue *from H. cinerascens* over summer and winter. Intercepts for the permutation validation were: R^2 = (0.0; 1) and Q^2 = (0.0; 1)

Supplementary Table 3. CV-ANOVA result from *H. cinerascens* gonadal tissue comparison over summer and winter.

M3	SS	DF	MS	F	p	SD
**Total corr.**	5	5	1			1
**Regression**	5	4	1,25	3,14146e+12	4,23151e-07	1,11803
**Residual**	3,97904e-13	1	3,97904e-13			6,30796e-07

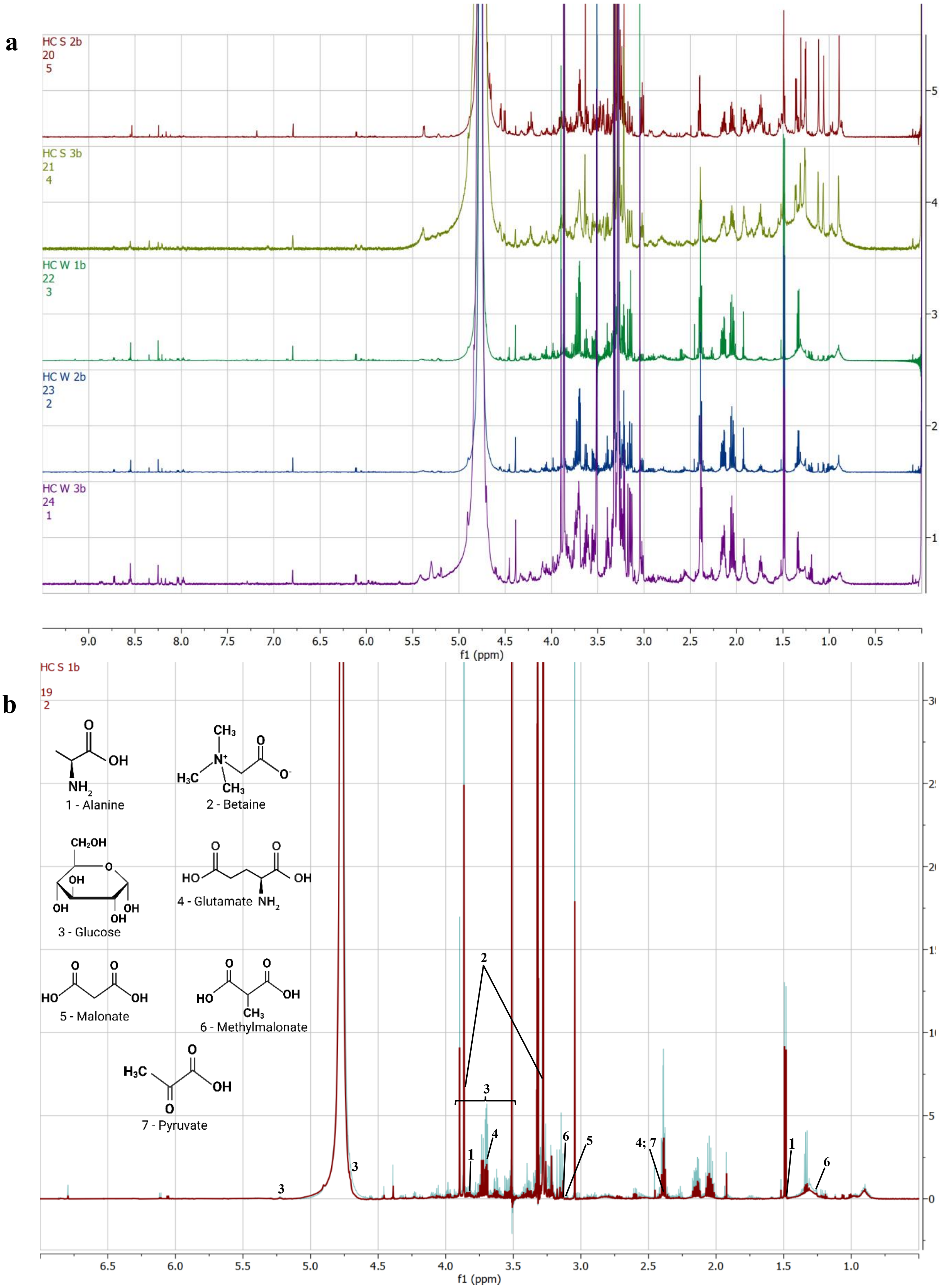

Supplementary Figure 6. (a) NMR spectral stack showing the chemical shift regions from the gonadal tissue comparison of *H. cinerascens* between summer ("S") and winter ("W"). **(b)** Labelled NMR spectral overlay of *"HC_S_1b"* and *"HC_W_1b"* samples reflecting key differentiating metabolites among summer (red) and winter (blue) gonadal tissue extracts. 1 – alanine, 2 – betaine, 3 – glucose, 4 – glutamate, 5 – malonate, 6 – methylmalonate, 7 – pyruvate. Structures created in BioRender. Okpeku, M. (2025) https://BioRender.com/173hkl5

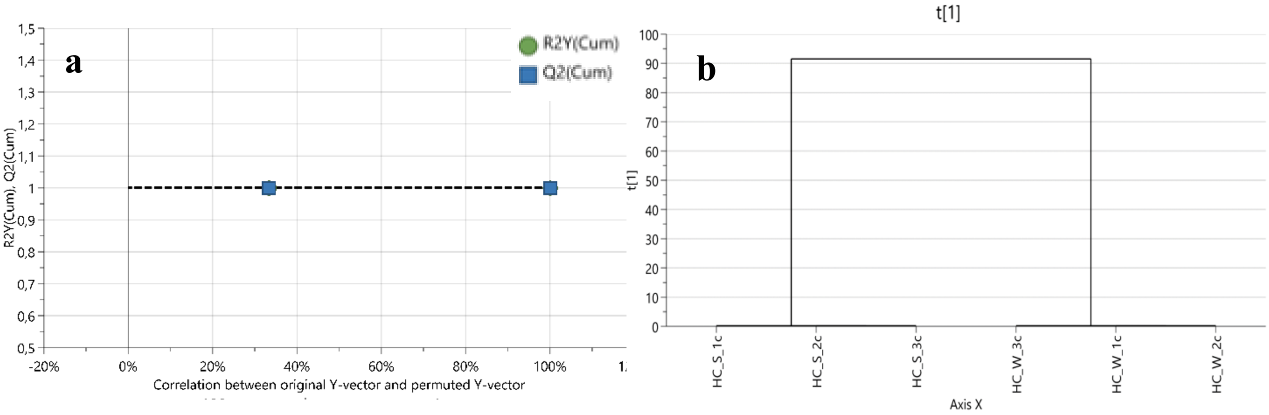

Supplementary Figure 7. Permutation validation (a) and Hierarchical Cluster Analysis (HCA) (b) for the gut/mesentery tissue from *H. cinerascens* over summer and winter. Intercepts for the permutation validation were: $R^2 = (0.0; 1)$ and $Q^2 = (0.0; 1)$.

Supplementary Table 4. CV-ANOVA result from *H. cinerascens* gut & mesentery tissue comparison over summer and winter.

M3	SS	DF	MS	F	p	SD
**Total corr.**	5	5	1			1
**Regression**	5	4	1,25	7,18048e+12	2,79888e-07	1,11803
**Residual**	1,74083e-13	1	1,74083e-13			4,17233e-07

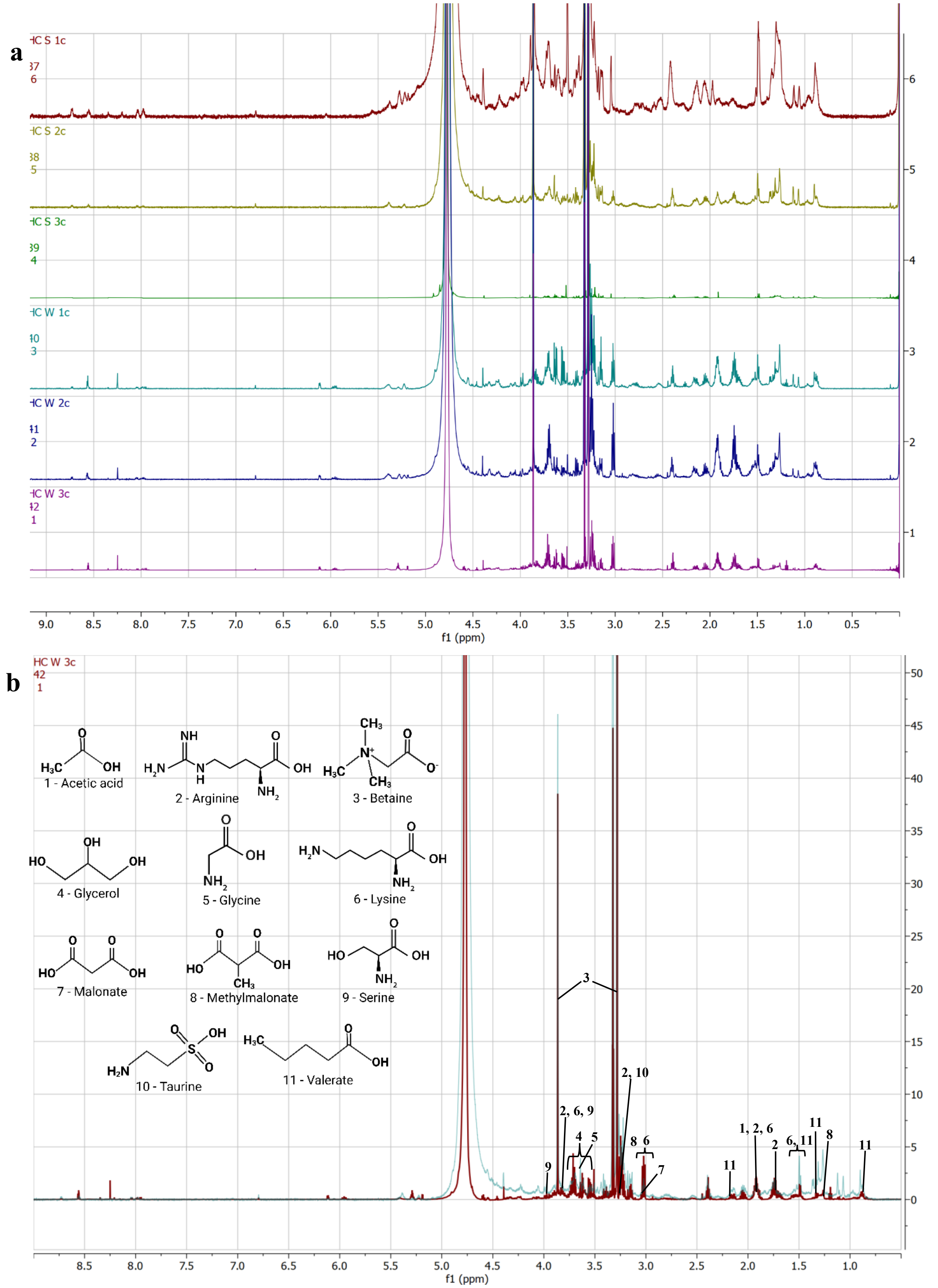

Supplementary Figure 8. (a) NMR spectral stack showing chemical shifts from the gut/mesentery comparison from *H. cinerascens* between summer ("S") and winter ("W"). **(b)** Labelled NMR spectral overlay of *"HC_S_2c"* and *"HC_W_3c"* samples reflecting key differentiating metabolites among summer (blue) and winter (red) gut/mesentery tissue extracts. 1 – acetic acid, 2 – arginine, 3 – betaine, 4 – glycerol, 5 – glycine, 6 – lysine, 7 – malonate, 8 – methylmalonate, 9 – serine, 10 – taurine, 11 – valerate. Structures created in BioRender. Okpeku, M. (2025) https://BioRender.com/jvee7mn

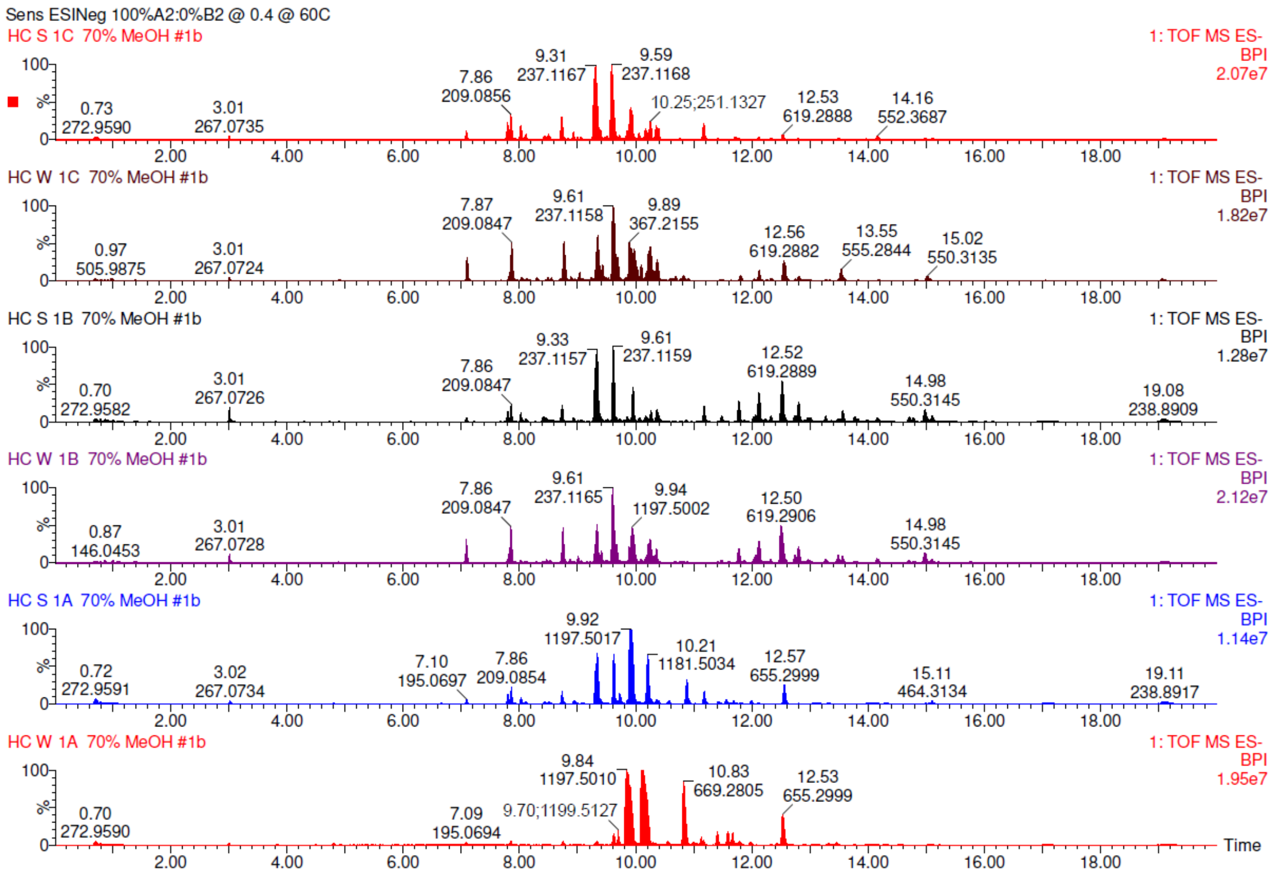

Supplementary Figure 9. UPLC-QTOF-MS spectral stack comparing the body tissues from *H. cinerascens* over summer and winter. With the letters "a" representing body wall, "b" = gonad, "c" = gut/mesentery tissues, "S" = summer and "W" = winter.

References

[1] N. K. Z. Zolkeflee, P. L. Wong, M. Maulidiani, N. S. Ramli, A. Azlan, A. Mediani, C. L. Tham, F. Abas, "Revealing metabolic and biochemical variations via 1H NMR metabolomics in streptozotocin-nicotinamide-induced diabetic rats treated with metformin." *Biochem. Biophys. Res. Commun.* **2024**, *708*, 149778. https://doi.org/10.1016/j.bbrc.2024.149778

[2] S. P. Mdlalose, M. Raletsena, K. Ntushelo, O. Bodede, D. M. Modise, "1H-NMR-Based Metabolomic Study of Potato Cultivars, Markies and Fianna, Exposed to Different Water Regimes." *Front. Sustain. Food Syst.* **2022**, *6*. https://doi.org/10.3389/fsufs.2022.801504

[3] U. K. Sundekilde, L. B. Larsen, H. C. Bertram, "NMR-Based Milk Metabolomics." *Metabolites.* **2013**, *3*, 204–222. https://doi.org/10.3390/metabo3020204

[4] A. P. Tikunov, C. B. Johnson, H. Lee, M. K. Stoskopf, J. M. Macdonald, "Metabolomic investigations of American oysters using H-NMR spectroscopy." *Mar. Drugs.* **2010**, *8*, 2578–2596. https://doi.org/10.3390/md8102578

[5] F. Bhinderwala, H. E. Roth, M. Filipi, S. Jack, R. Powers, "Potential Metabolite Biomarkers of Multiple Sclerosis from Multiple Biofluids." *ACS Chem. Neurosci.* **2024**, *15*, 1110–1124. https://doi.org/10.1021/acschemneuro.3c00678

[6] J. Oh, D. H. Yoon, J. G. Han, H. K. Choi, G. H. Sung, "(1)H NMR based metabolite profiling for optimizing the ethanol extraction of *Wolfiporia cocos*." *Saudi J. Biol. Sci.* **2018**, *25*, 1128–1134. https://doi.org/10.1016/j.sjbs.2018.04.007

[7] M. T. Akhtar, M. Samar, A. A. Shami, M. W. Mumtaz, H. Mukhtar, A. Tahir, S. Shahzad-Ul-Hussan, S. U. Chaudhary, U. Kaka, "1H-NMR-Based Metabolomics: An Integrated Approach for the Detection of the Adulteration in Chicken, Chevon, Beef and Donkey Meat." *Molecules.* **2021**, *26*, 4643. https://doi.org/10.3390/molecules26154643

[8] P. F. Sehlakgwe, N. Lall, G. Prinsloo, "1H-NMR Metabolomics and LC-MS Analysis to Determine Seasonal Variation in a Cosmeceutical Plant *Leucosidea sericea*." *Front. Pharmacol.* **2020**, *11*, 219. https://doi.org/10.3389/fphar.2020.00219

[9] G.-Q. Sun, L. Li, Y.-H. Yi, W.-H. Yuan, B.-S. Liu, Y.-Y. Weng, S.-L. Zhang, P. Sun, Z.-L. Wang, "Two New Cytotoxic Nonsulfated Pentasaccharide Holostane (=20-Hydroxylanostan-18-oic Acid γ-Lactone) Glycosides from the Sea Cucumber *Holothuria grisea*." *Helv. Chim. Acta.* **2008**, *91*, 1453–1460. https://doi.org/10.1002/hlca.200890158

[10] M. Xing, F. Liu, J. Lin, D. Xu, J. Zhong, F. Xia, J. Feng, G. Shen, "Origin tracing and adulteration identification of bird's nest by high- and low-field NMR combined with pattern recognition." *Food Res. Int.* **2024**, *175*, 113780. https://doi.org/10.1016/j.foodres.2023.113780

[11] N. Nkobole, G. Prinsloo, "1H-NMR and LC-MS Based Metabolomics Analysis of Wild and Cultivated *Amaranthus* spp." *Molecules.* **2021**, *26*, 795. https://doi.org/10.3390/molecules26040795

[12] W. M. Bandaranayake, A. D. Rocher, "Role of secondary metabolites and pigments in the epidermal tissues, ripe ovaries, viscera, gut contents and diet of the sea cucumber *Holothuria atra*." *Mar. Biol.* **1999**, *133*, 163–169. 10.1007/s002270050455

[13] F. Angilè, L. Del Coco, C. R. Girelli, L. Basso, L. Rizzo, S. Piraino, L. Stabili, F. P. Fanizzi, "1H NMR Metabolic Profile of *Scyphomedusa Rhizostoma pulmo* (Scyphozoa, Cnidaria) in Female Gonads and Somatic Tissues: Preliminary Results." *Molecules.* **2020**, *25*, 806. 10.3390/molecules25040806

[14] G. K. More, S. Meddows-Taylor, G. Prinsloo, "Metabolomic Profiling of Antioxidant Compounds in Five *Vachellia* Species." *Molecules.* **2021**, *26*, 6214. https://doi.org/10.3390/molecules26206214
